# Rare disorders have many faces: in silico characterization of rare disorder spectrum

**DOI:** 10.1186/s13023-022-02217-9

**Published:** 2022-02-22

**Authors:** Simona D. Frederiksen, Vladimir Avramović, Tatiana Maroilley, Anna Lehman, Laura Arbour, Maja Tarailo-Graovac

**Affiliations:** 1 Departments of Biochemistry, Molecular Biology and Medical Genetics, Cumming School of Medicine, University of Calgary, Calgary, AB T2N 4N1 Canada; 2 Alberta Children’s Hospital Research Institute, University of Calgary, Calgary, AB T2N 4N1 Canada; 3 Department of Medical Genetics, University of British Columbia, Vancouver, BC V6T 1Z2 Canada

**Keywords:** Borderline-common, Diagnostics, Pipelines, Developmental defect, Neurological, Causative genes, Phenotypes, Pathway analysis, Orphanet

## Abstract

**Background:**

The diagnostic journey for many rare disease patients remains challenging despite use of latest genetic technological advancements. We hypothesize that some patients remain undiagnosed due to more complex diagnostic scenarios that are currently not considered in genome analysis pipelines. To better understand this, we characterized the rare disorder (RD) spectrum using various bioinformatics resources (e.g., Orphanet/Orphadata, Human Phenotype Ontology, Reactome pathways) combined with custom-made R scripts.

**Results:**

Our in silico characterization led to identification of 145 borderline-common, 412 rare and 2967 ultra-rare disorders. Based on these findings and point prevalence, we would expect that approximately 6.53%, 0.34%, and 0.30% of individuals in a randomly selected population have a borderline-common, rare, and ultra-rare disorder, respectively (equaling to 1 RD patient in 14 people). Importantly, our analyses revealed that (1) a higher proportion of borderline-common disorders were caused by multiple gene defects and/or other factors compared with the rare and ultra-rare disorders, (2) the phenotypic expressivity was more variable for the borderline-common disorders than for the rarer disorders, and (3) unique clinical characteristics were observed across the disorder categories forming the spectrum.

**Conclusions:**

Recognizing that RD patients who remain unsolved even after genome sequencing might belong to the more common end of the RD spectrum support the usage of computational pipelines that account for more complex genetic and phenotypic scenarios.

**Supplementary Information:**

The online version contains supplementary material available at 10.1186/s13023-022-02217-9.

## Background

Rare disease diagnostic timeliness and accuracy are still suboptimal, in spite of technological inventions of the twenty-first century. High-throughput sequencing has led to considerable progress in rare disease diagnostics and discovery [1], yet it takes on average eight years to get accurately diagnosed with a couple of misdiagnoses along the way [2]. For some patients it has even taken up to 30 years to get an etiologic diagnosis while others remain throughout their life without a definite diagnosis [3]. Gilissen and colleagues reported a 42% diagnostic rate using whole genome sequencing and a trio-based design (based on 50 patients with severe intellectual disability and their unaffected parents) [4–6]. There are numerous reasons why health professionals are unable to establish accurate diagnoses for the patients under investigation. A source of diagnostic errors is ‘no-fault errors’ which can arise if the disease representation is atypical or masked [7, 8]. Incomplete penetrance and variable expressivity (sometimes referred to as non-Mendelian phenomena) are believed to be the underlying factors behind this observation [9–11]. Other examples of factors that can result in diagnostic errors are low read depth in the genetic region of interest, relatively high allele frequencies in a reference population, existence of allelic imbalance/mosaicism, the causative variant being inherited from an unaffected parent, faulty pathogenicity predictions using computational tools, or the condition not being genetically inherited (e.g. arise from imprinting) [12].

This is where the concept of missing heritability comes in—often used in the context of common disease. Missing heritability refers to the fraction of heritability that we have not yet been able to explain using state-of-the-art methodologies. Kong stated that *“it is reasonable to assume that complex inheritance as a whole could account for a substantial fraction of heritability”* [13]. This was discussed by Maroilley and Tarailo-Graovac in 2019 but focusing on rare disease [14]. Approximately 80% of rare diseases are estimated to have a genetic origin. These rare diseases may suffer from missing heritability [15, 16]. One question worth asking is, *how can we explain the missing heritability that might be the cause of the challenges we are facing in rare disease diagnostics?* Some of the missing heritability might be explained by more complex genetic scenarios such as the implication of structural and other complex variants or multigenic inheritance of rare diseases rather than the conservative ‘one-causative-gene’-approach. By considering more complex genetic scenarios in computational pipelines focused on rare disease diagnostics, we might be able to explain some of the missing heritability. Additionally, utilization of phenomics (the acquiring of high-dimensional phenotypic data) [17] could also be useful in addressing variable expressivity and incomplete penetrance as well as their impact on diagnostic rates. Computational pipelines are not currently geared to address more complex genetic and phenotypic scenarios, and negate the whole palette of rare diseases (e.g., some rare diseases are more common than others) and thus the underlying genetic architecture might differ. This consideration is based on our knowledge that common diseases are considered polygenic and multifactorial.

Mendelian diseases are diseases *“for which alternative genotypes fall into distinct, discrete phenotypic classes, following Gregor Mendel’s laws of inheritance”* [18], which mostly concern monogenic rare diseases [19]. For these rare diseases, we expect strong penetrance and invariable expressivity in contrast to the multifactorial common diseases. However, these disease groups represent two broad categories rather than all diseases along the spectrum. Importantly, researchers have started to pay more attention to digenic and oligogenic inheritance underlying rare diseases as well as interactions and genetic modifiers over the past decade [20–24]. This has led to many new discoveries and developments such as the DIgenic diseases DAtabase (DIDA) [25], the Oligogenic Resource for Variant AnaLysis (ORVAL) [26], an interactome-based platform [27], and the genetic modifier database, PhenoModifier [28]. Focusing on well-established resources, DatabasE of genomiC varIation and Phenotype in Humans using Ensembl Resource (DECIPHER) [29], genome aggregation database (gnomAD) [30] and ClinVar [31] have greatly benefited health professionals working with rare disease diagnostics and therefore the patients who seek clarity regarding their health concerns. Nevertheless, the analytical methods available have various limitations [32], which might delay an accurate diagnosis and optimal treatment. Another potential reason for diagnostic failure is that those methods have been developed generally with the purpose of diagnosing either rare or common diseases rather than considering the entire disease spectrum.

This brings up the question: *Where does the line go between rare and common?* Even though it may seem like a simple question, it has caused a lot of debate. The most widely accepted international definitions for a rare disease in terms of prevalence are (1) less than 1 in 2000 people in the European Union (https://ec.europa.eu/), and (2) less than 200,000 people in the United States of America (defined in the Orphan Drug Act). Nevertheless, Richter et al. [33] identified almost 300 different rare disease definitions from various organizations and found that the most broadly used prevalence threshold was 40–50 cases per 100,000 people. Moreover, the prevalence for ultra-rare diseases has been reported to be less than 1 case per 1,000,000 people [34]. We used these thresholds as pin pointers to dissect the range of the disorder spectrum considered as rare, and refer to rare disorders as borderline-common, rare, and ultra-rare (a spectrum ranging from a point prevalence of 6–9/10,000 to < 1/1,000,000). More recently, focus has changed from asking *“Is it rare?”* to *“How rare is it?”* as phrased by Jason et al. [35] Therefore, the overall aim of this study was to characterize the RD spectrum. We expect that understanding the characteristics of disorders across the spectrum will help us to categorize ‘difficult-to-diagnose’ patients to specific disorder categories, which can provide guidance on the selection of most appropriate analytical methods for the patient under investigation. This is of particular importance in the Silent Genomes where many of the enrolled Indigenous patients with suspected genetic conditions remain undiagnosed even after whole genome sequencing, as well as for other ‘difficult-to-diagnose’ RD patients. We hypothesize that multiple undiagnosed patients with rare conditions belong to the less rare end of the RD spectrum and thus their phenotype representations can be explained by more complex genetic scenarios (e.g., it is well known that common disorders are multigenic/multifactorial).

## Results

**Fig. 1:**
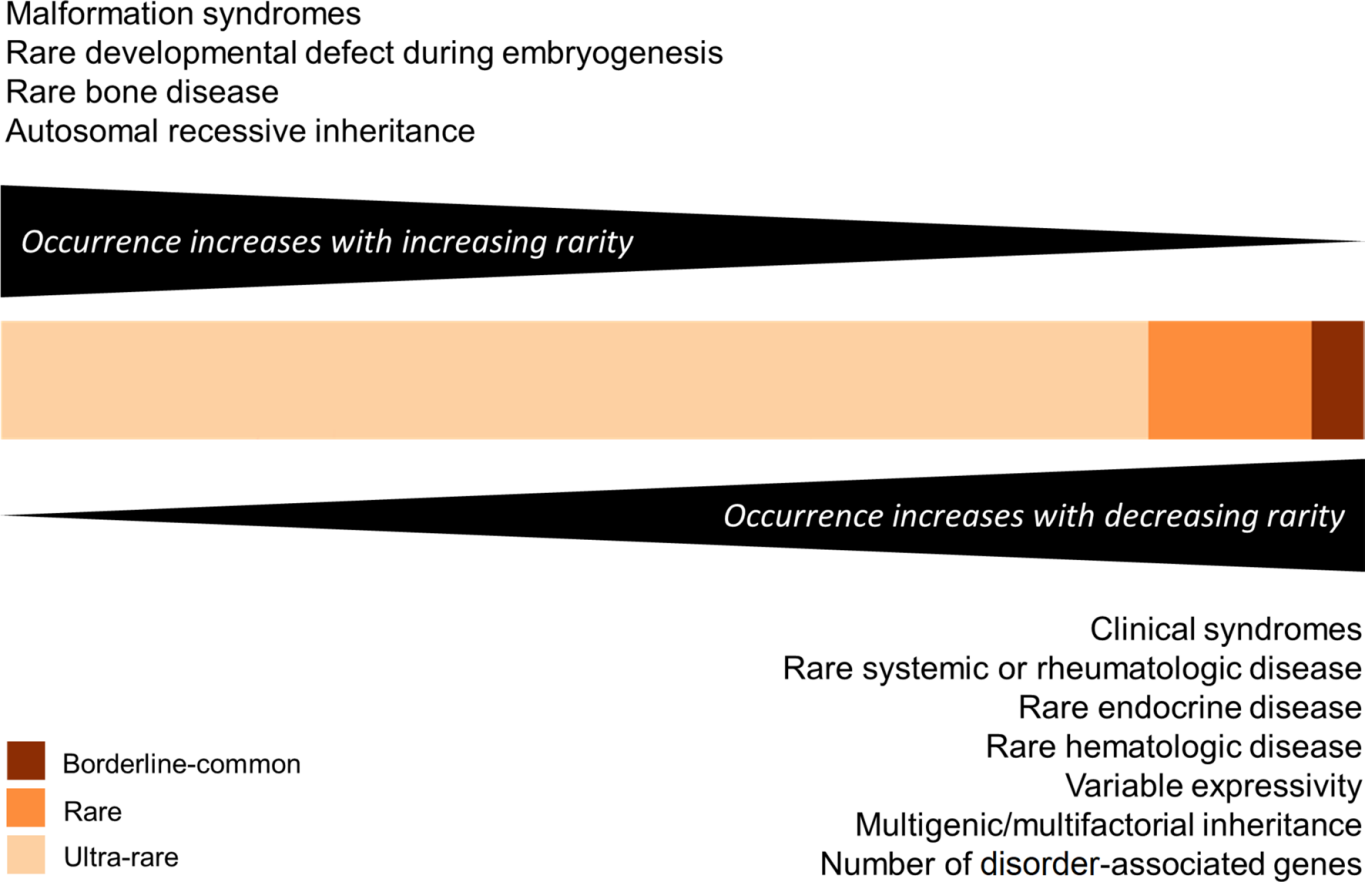
Summary of findings for the rare disorder spectrum

Our characterization of the RD spectrum, focusing on a wide range of factors ranging from disorder types to associated HPO terms and genes, is summarized in Fig. 1.

### Borderline-common disorders only comprise 4% of disorders in the spectrum yet represented more than 90% of patients in a fictive rare disorder cohort

Worldwide and/or continent point prevalence were reported for 3,524 RDs in Orphadata (Additional File 1: Fig. S1). By focusing on worldwide and continent point prevalence, we believe that the issue of founder and consanguinity effects were eliminated. In agreement, none of the included RDs had a prevalence of more than 1 case per 1,000 people (one of the prevalence categories in Orphanet), which mainly was observed in specific regions or for specific populations (e.g., French Canadians of Quebec, Canadian Indigenous Peoples).

**Fig. 2:**
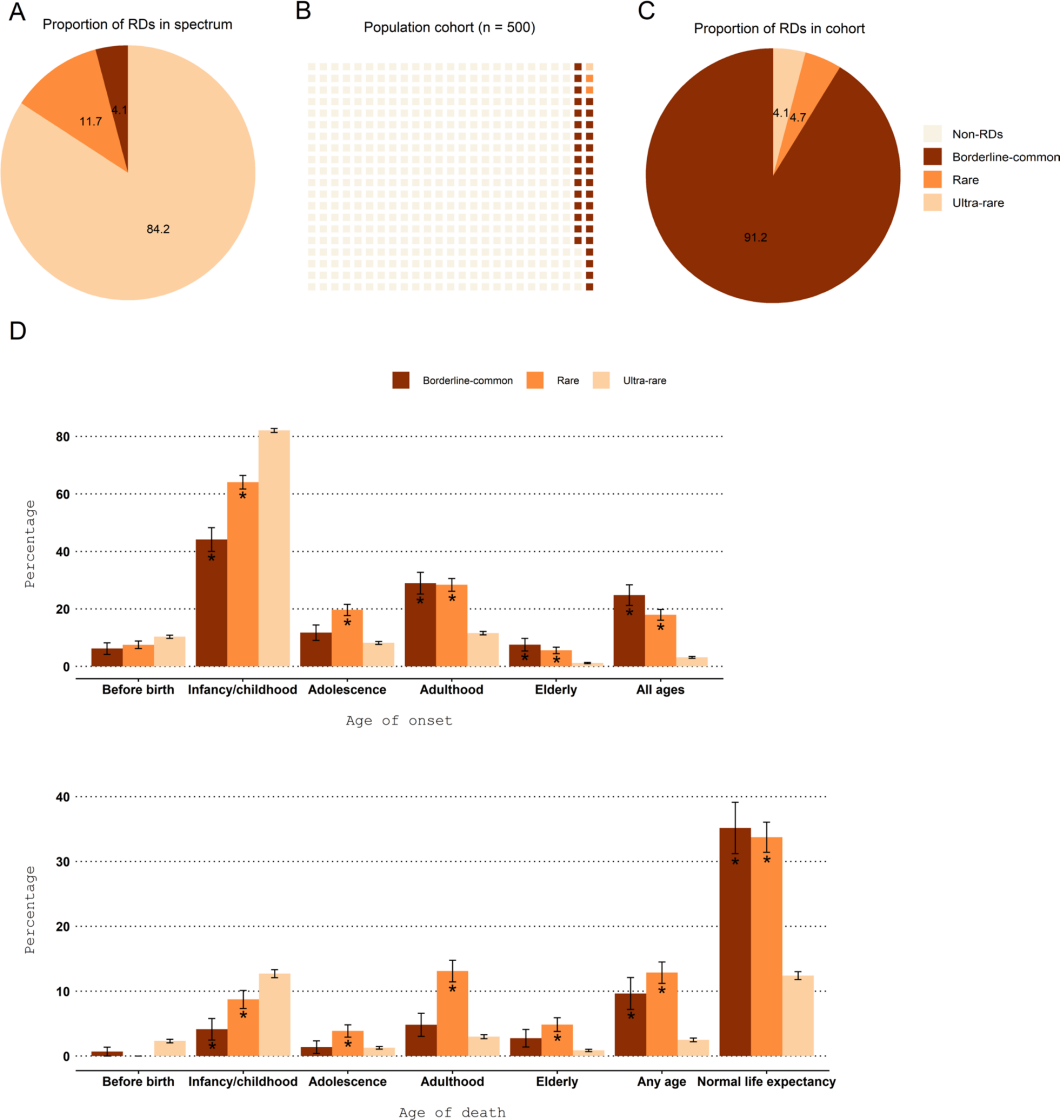
Prevalence and natural history of borderline-common, rare, and ultra-rare disorders. **A** We identified 3,524 rare disorders (RDs) in the spectrum of which 4.1%, 11.7% and 84.2% belonged to the borderline-common, rare, and ultra-rare disorder category, respectively. **B** Prevalence of borderline-common, rare, and ultra-rare disorders in a randomly selected population. If taking point prevalence (midpoint) and number of RDs for each disorder category into consideration, this would result in 6.5%, 0.34% and 0.30% of individuals in a randomly selected population to have a borderline common, rare, and ultra-rare disorder, in turn. **C** If focusing solely on the group of RD patients within the population presented in Fig. 2B, 91.2% of them would have a borderline-common disorder. **D** The interval average age of onset and death for RDs within the spectrum were represented as proportions and standard errors. In total, 96.3% of the RDs had information about interval average age of onset, and 64.2% of the RDs had information about interval average age of death. Note that the percentages were calculated based on total count of RDs within each disorder category, as presented in Additional File 1: Fig. S1. For each level of age of onset (6 levels) and age of death (7 levels), we examined if the proportions for the borderline-common and rare disorder categories differed significantly from the ultra-rare disorder category and marked significant findings (*)

Most RDs belonged to the ultra-rare disorder category (RD = 2967, 84.2%) followed by the disorder categories rare (RD = 412, 11.7%) and borderline-common (RD = 145, 4.1%; Fig. 2A). When considering both point prevalence (midpoint) and number of RDs, it translates into approximately 0.30%, 0.34%, and 6.5% of them having an ultra-rare, rare, and borderline-common disorder in a randomly selected population, respectively (Fig. 2B). Thus, it is expected that 33 people have a borderline-common disorder, 2 people have a rare disorder and 1 person has an ultra-rare disorder in a population of 500 randomly selected people (Fig. 2B). This equals to 1 in 14 people living with a RD (i.e., 500/36 ≈ 14), all categories considered. By focusing solely on the predicted RD patients within this fictive population, we can appreciate that borderline-common disorders are more prevalent than rare and ultra-rare disorders and may represent a substantial portion of an undiagnosed cohort (Fig. 2C, Additional File 2: Table S2).

### A higher proportion of borderline-common disorders have a more complex genetic inheritance than rare and ultra-rare disorders

Understanding how disorders are inherited can provide us with information about how to tailor our diagnostic computational pipelines. Assigning inheritance type to the disorders in Orphanet is based on evidence and expert knowledge. Using Orphadata, we identified nine different inheritance types (Table 1). Across the RD spectrum, the proportion of RDs caused by autosomal recessive inheritance increased with increasing rarity, and the proportion of RDs caused by multigenic/multifactorial inheritance decreased with rarity. Interestingly, oligogenic inheritance was reported only for one RD within the rare disorder category, namely Bardet-Biedl syndrome [Orphacode:110]. We also found significantly more RDs within the rare disorder category caused by autosomal dominant inheritance when compared with the ultra-rare disorder category. Nevertheless, autosomal recessive inheritance (20.0–42.5%) and autosomal dominant inheritance (25.5–32.3%) were the most common types of inheritance for each of the disorder categories. The proportion of ultra-rare disorders inherited in an autosomal recessive pattern was more than double the proportion of borderline-common disorders which could provide one explanation for the differences in disorder prevalence. For the borderline-common disorders, multigenic/ multifactorial inheritance also played a considerable role (14.5%) (Table 1).

**Table 1:** Summary of disorder types, linearisation parents and type of inheritance for the rare disorder spectrum

Category	Overall^a^	Borderline-common		Rare		Ultra-rare	D^b^
Measures^c^	Prop, %	Prop, %	Adj. *p*-value^d^	Prop, %	Adj. *p*-value^d^	Prop, %	
***Disorder type***							
Disease	58.14	61.38	NS	76.70	1.06 × 10^–16^	55.41	–
Malformation syndrome	38.17	11.03	1.69 × 10^–15^	15.78	2.14 × 10^–27^	42.60	↑
Morphological anomaly	2.36	11.72	4.13 × 10^–09^	4.37	9.97 × 10^–04^	1.62	↓
Particular clinical situation in a disease or syndrome	0.88	13.79	6.32 × 10^–26^	2.43	1.08 × 10^–08^	0.03	↓
Clinical syndrome	0.31	2.07	5.84 × 10^–03^	0.73	NS	0.17	↓
Biological anomaly	0.14	–	–	–	–	0.17	–
***Top 10 linearisation parents for the RD spectrum***							
Rare developmental defect during embryogenesis	37.97	21.38^e^	7.75 × 10^–06^	21.12^e^	1.17 × 10^–14^	41.12^e^	↑
Rare neurologic disease	20.09	8.97^e^	9.59 × 10^–04^	22.82^e^	NS	20.26^e^	–
Rare inborn errors of metabolism	7.63	1.38	4.25 × 10^–03^	11.89^e^	9.34 × 10^–03^	7.35^e^	–
Rare bone disease	7.49	1.38	1.68 × 10^–03^	4.37	1.05 × 10^–02^	8.22^e^	↑
Rare skin disease	5.56	5.52	NS	5.58^e^	NS	5.56^e^	–
Rare ophthalmic disorder	3.41	8.28^e^	3.48 × 10^–03^	5.10	4.64 × 10^–02^	2.93	↓
Rare immune disease	3.15	0.69	NS	3.16	NS	3.27	–
Rare endocrine disease	2.95	6.90	7.76 × 10^–03^	4.85	2.13 × 10^–02^	2.49	↓
Rare hematologic disease	2.89	8.28^e^	8.25 × 10^–04^	5.10	9.34 × 10^–03^	2.33	↓
Rare systemic or rheumatologic disease	2.67	10.34^e^	2.31 × 10^–06^	6.31^e^	7.32 × 10^–06^	1.79	↓
***Type of inheritance***							
Autosomal recessive	41.23	20.00	1.31 × 10^–07^	39.81	NS	42.47	↑
Autosomal dominant	26.39	28.97	NS	32.28	1.63 × 10^–02^	25.45	–
X-linked recessive	6.47	4.14	NS	8.74	NS	6.27	–
X-linked dominant	1.45	0.69	NS	1.94	NS	1.42	–
Mitochondrial inheritance	0.54	1.38	NS	1.21	NS	0.40	–
Semi-dominant	0.11	0.69	NS	–	–	0.10	–
Y-linked	0.06	0.69	NS	–	–	0.03	–
Oligogenic	0.03	–	–	0.24	–	–	–
Multigenic/multifactorial	1.59	14.48	2.04 × 10^–21^	6.31	1.67 × 10^–16^	0.30	↓

The rare disorder (RD) spectrum consists of 3,524 RDs of which 145, 412 and 2,967 RDs were categorized as borderline-common, rare, and ultra-rare, respectively. The proportions within the borderline-common and rare disorder categories were compared with those within the ultra-rare disorder category

^a^No RDs were categorized into the > 1/1000 prevalence group

^b^Direction of change of proportions from borderline-common to ultra-rare (D)

^c^Proportion (prop) was calculated based on total number of RDs within the disorder category

^d^FDR-adjusted *p* < 0.05 was considered statistically significant; NS refers to not significant

^e^Top 5 linearisation parents within the disorder category

The genes associated with the disorders are based on findings from peer-reviewed publications and biomarker testing practices, as carried out by Orphanet [36]. The genes reported in Orphadata [37] are not only disease-causing but also modifying or known to alter the susceptibility of the disorder. The number of genes associated with specific RDs increase, on average, with decreasing rarity [reported as median (IQR), 3 (6) causative genes for borderline-common disorders; 1 (2) causative genes for rare disorders; 1 (0) causative genes for ultra-rare disorders]. Focusing on associated genes, the mean ranks were significantly higher for borderline-common and rare disorders as compared with ultra-rare disorders (Table 2), indicating more associated genes for these disorder categories.

**Table 2:** Occurrence of Human Phenotype Ontology (HPO) terms and disorder-associated genes across the rare disorder spectrum

Category	Overall	Borderline-common		Rare		Ultra-rare	D^a^
Measures	Median	Median (IQR)	Adj. *p*-value^b^	Median (IQR)	Adj. *p*-value^b^	Median (IQR)	
***Disorder-associated genes per disorder***							
Associated genes	1	3 (6)	< 2.0 × 10^–16^	1 (2)	< 2.0 × 10^–16^	1 (0)	↓
***HPO terms per disorder***^c^							
Count, HPO term_total_	20	17 (22)	NS	24 (24)	1.9 × 10^–06^	19 (19)	–
Count, HPO term_obligate/very frequent_	6	4 (25)	1.4 × 10^–06^	6 (8)	NS	6 (8)	–
Count, HPO term_occasional/very rare_	8	9 (15)	4.1 × 10^–02^	10 (14)	2.4 × 10^–06^	7 (12)	–
Ratio, HPO term _(obligate/very frequent)/total_	0.31	0.22 (0.36)	3.7 × 10^–03^	0.25 (0.33)	1.5 × 10^–04^	0.33 (0.56)	↑
Ratio, HPO term _(occasional/very rare)/total_	0.22	0.44 (0.38)	1.5 × 10^–12^	0.36 (0.38)	< 2.0 × 10^–16^	0.18 (0.42)	↓

A total of 2,430 rare disorders (RDs) had associated HPO terms based on Orphadata. Here, we provide counts of HPO terms for the rare disorders (RD) within the spectrum, including the ratio between them (the latter as measures of phenotypic expressivity). The measures of phenotypic expressivity are based on the HPO term occurrence within RDs. We also provide count of genes associated with the RDs. The mean ranks within the borderline-common (RD = 131) and rare (RD = 387) disorder categories were compared with those within the ultra-rare (RD = 1,912) disorder category; and the distributions are illustrated in Additional File 1: Fig. S5

^a^Direction of change of proportions from borderline-common to ultra-rare (D)

^b^FDR-adjusted *p* < 0.05 was considered statistically significant; NS refers to not significant

^c^HPO term occurrence: Obligate (100%), very frequent (99–80%), occasional (29–5%), very rare (< 4–1%). The percentages indicate how many patients with a certain RD who are expected to have the HPO term in question

### Borderline-common and rare disorders tend to be less life threatening and a higher proportion of those develop later in life than ultra-rare disorders

During the diagnostic process, knowledge about interval average age of onset and interval average age of death can help us to (1) exclude disorders without relevance to our patient assessment, (2) be informed about long-term survival prospects for the patients under investigation, and (3) identify late onset disorders where predictions, addressing who is at risk, can be beneficial. Using Orphadata [37], more RDs within the spectrum had information about interval average age of onset than interval average age of death hence the differences in percentage magnitude in Fig. 2D between the two variables. It is also important to consider that information about interval average age of death were only available for 64.2% of RDs in the spectrum.

Focusing on interval average age of onset (Fig. 2D), most RDs develop during infancy (4 weeks–23 months) or childhood (2–11 years) compared with the other age groups but is most pronounced for the ultra-rare disorders. A higher proportion of RDs within the borderline-common and rare disorder categories develop during adulthood (19–65 years), in elderly (after 65 years), and at all ages (from birth to adulthood) when compared with the ultra-rare disorder category. During adolescence (12–18 years), a higher proportion of RDs within the rare disorder category arise compared with the ultra-rare disorder category.

Focusing on interval average age of death (Fig. 2D), many RD patients can expect a normal life expectancy, especially those with a borderline-common or rare disorder. For example, a higher proportion of patients with a borderline-common or rare disorder have a normal life expectancy in relation to those with an ultra-rare disorder (Fig. 2). For a considerable proportion of ultra-rare disorders, death occurs during infancy or childhood. For the rare disorder category, death occurs more frequently during adolescence, in adulthood or in elderly when compared with the other disorder categories. Death occurring at any age has been reported for a larger proportion of borderline-common and rare disorders than ultra-rare disorders.

**Fig. 3:**
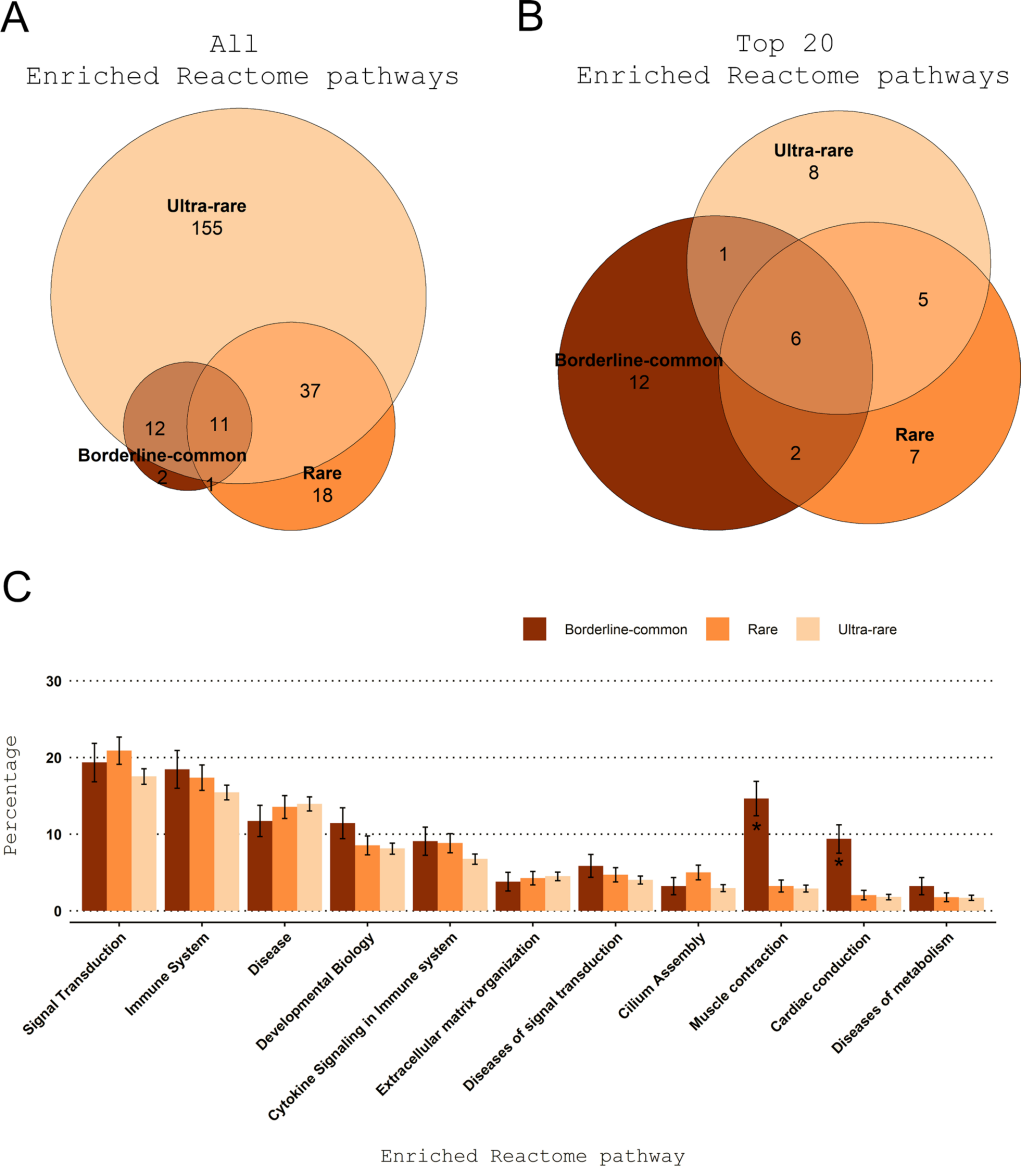
Enriched Reactome pathways for the borderline-common, rare, and ultra-rare disorders. **A**, **B** The Venn diagrams illustrates the overlapping and non-overlapping enriched Reactome pathways between the borderline-common, rare, and ultra-rare disorder categories focusing on all and top 20 enriched Reactome pathways (Fig. 4). **C** Overview of the proportion (and standard error) of disorder-associated genes annotated to the enriched Reactome pathways in relation to the total number of disorder-associated genes for the disorder category in question. Eleven enriched Reactome pathways were found for each of the disorder categories

**Fig. 4:**
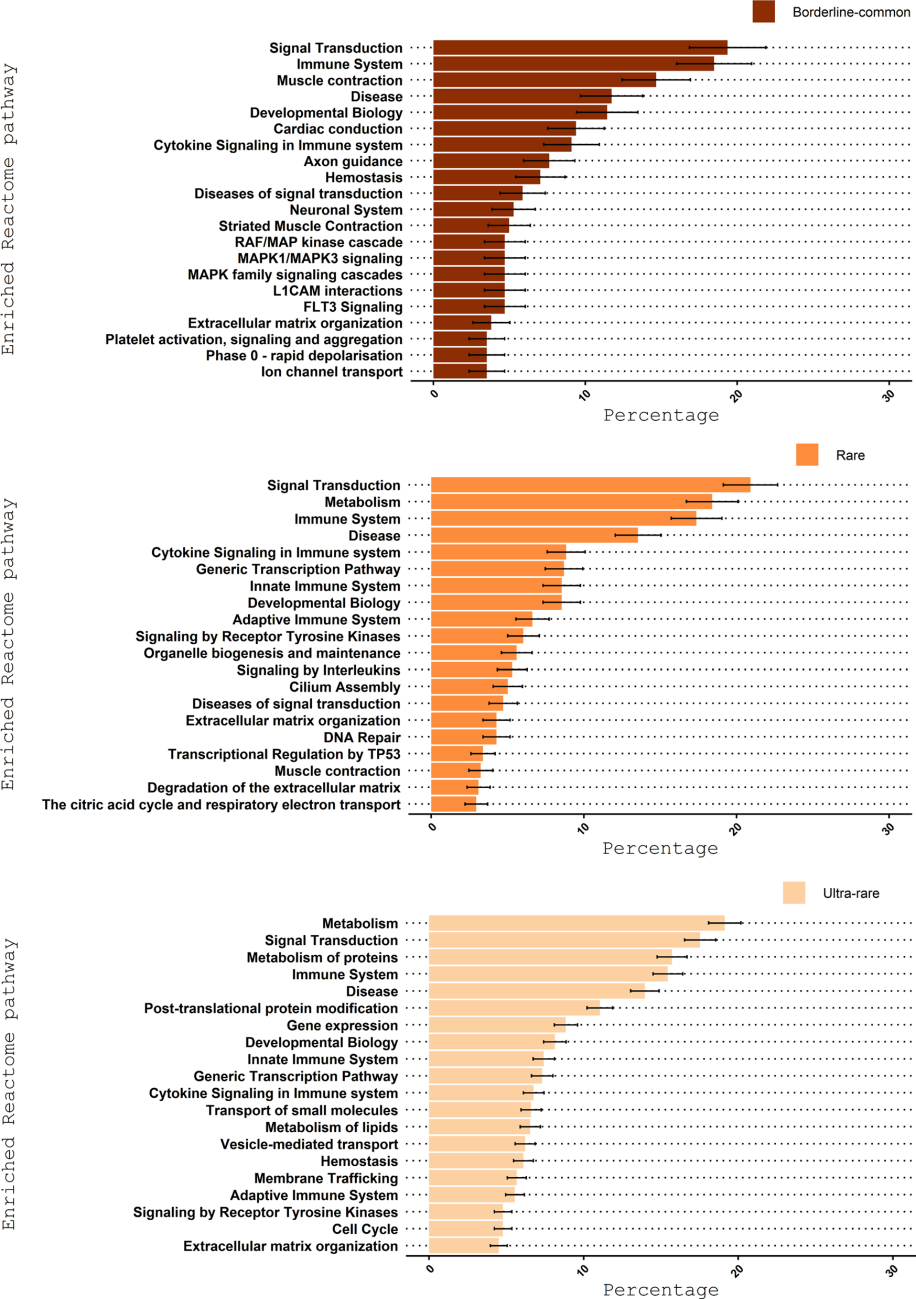
Top 20 enriched Reactome pathways for the borderline-common, rare, and ultra-rare disorders. For the top 20 enriched Reactome pathways, 6 enriched Reactome pathways (disease, developmental biology, signal transduction, immune system, cytokine signaling in immune system and extracellular matrix organization) were found for each of the disorder categories. In addition, diseases of signal transduction and muscle contraction were enriched for both the borderline-common and rare disorder category, and adaptive immune system, metabolism, generic transcription pathway, innate immune system and signaling by receptor tyrosine kinases were enriched for both the rare and ultra-rare disorder category within top 20

Interestingly, some of the enriched Reactome pathways unique to the ultra-rare disorder category are known to be essential for proper bodily functions (e.g., gene expression, translation, cell cycle; Figs. 3, 4). Our observation that pathways affecting essential bodily functions are involved in the development of some ultra-rare disorders agrees with our other finding that ultra-rare disorders are more life threatening.

### The clinical characteristics tend to vary across the rare disorder spectrum

**Fig. 5:**
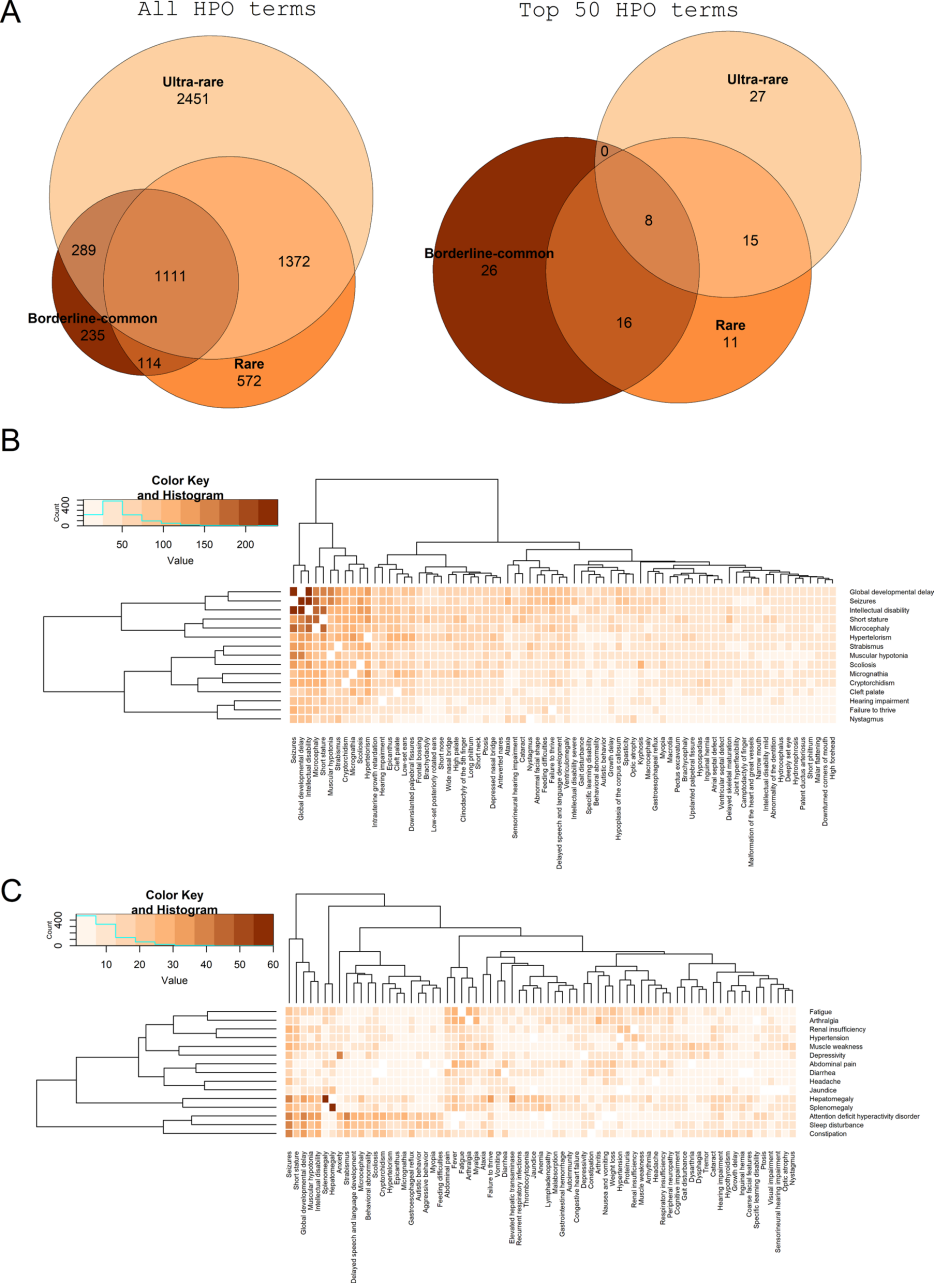
Human Phenotype Ontology (HPO) terms and co-occurrence profiles for the rare disorder spectrum. **A** The Venn diagrams illustrate similarities and differences in HPO terms across the disorder categories. As the number of rare disorders (RDs) categorized to a specific disorder category increase with decreasing prevalence, we expected to find more HPO terms for the ultra-rare disorder category. Therefore, we provided a Venn diagram for the top 50 HPO terms, in addition to the one representing all HPO terms. For the top 50 HPO terms, the disorder categories had 8 HPO terms in common, namely seizures, short stature, hearing impairment, scoliosis, cleft palate, global developmental delay, intellectual disability and muscular hypotonia. The rare disorder category shared a similar number of HPO terms with both the borderline-common and ultra-rare disorder category, yet these HPO terms did not overlap between the latter disorder categories. Focusing on all HPO terms, more HPO terms overlapped between the rare and ultra-rare disorder category compared with the borderline-common and ultra-rare disorder category. As differences between disorder categories appeared (e.g., multiple HPO terms unique to the disorder category), we further dissected the top findings (presented in Table 3). **B** Co-occurrence of the top 15 HPO terms within the RD spectrum (as shown in Table 3) with other HPO terms selected based on high relative co-occurrence. The HPO terms co-occurred in 3 to 239 RDs depending on the combination. **C** Co-occurrence of the top 15 HPO terms within the borderline-common disorder category (as represented in Table 3) with selected HPO terms based on high relative co-occurrence. Here, we decided to exclude the top 15 HPO terms, hearing impairment, seizures, short stature, and scoliosis, as they were also represented in Fig. 3B. Notably less co-occurrence between HPO terms was observed within this group (ranged from 1 to 60 RDs). Larger representations of this figure can be found in Additional File 1: Fig. S2-S3

Next, we explored the clinical characteristics of each of those categories. The rare disorder category appeared to be an intermediate of the ultra-rare and borderline-common disorder categories in multiple instances (Tables 1, 2, 3, Figs. 3B, 5A).

**Table 3:** Overview of Human Phenotype Ontology (HPO) terms across the rare disorder spectrum

Category	Overall	Borderline-common		Rare		Ultra-rare	D^a^
Measures^b^	Prop, %	Prop, %	Adj. *p*-value^c^	Prop, %	Adj. *p*-value^c^	Prop, %	
***Top 15 HPO terms for the RD spectrum***							
Seizures	22.92	16.79^d^	NS	23.77^d^	NS	23.17^d^	–
Intellectual disability	21.98	6.87	8.49 × 10^–05^	17.31^d^	2.85 × 10^–02^	23.95^d^	↑
Short stature	21.40	10.69^d^	1.14 × 10^–02^	17.31^d^	NS	22.96^d^	↑
Global developmental delay	20.04	6.87	4.41 × 10^–04^	13.44^d^	1.02 × 10^–03^	22.28^d^	↑
Microcephaly	15.68	2.29	1.06 × 10^–05^	6.20	1.32 × 10^–08^	18.51^d^	↑
Hypertelorism	15.06	3.82	3.22 × 10^–04^	6.72	6.05 × 10^–07^	17.52^d^	↑
Scoliosis	14.94	8.40^d^	NS	16.28^d^	NS	15.12^d^	–
Muscular hypotonia	13.09	6.87	NS	14.21^d^	NS	13.28^d^	–
Strabismus	12.76	4.58	1.75 × 10^–02^	9.82^d^	NS	13.91^d^	↑
Micrognathia	12.67	4.58	1.05 × 10^–02^	5.17	1.69 × 10^–06^	14.75^d^	↑
Cryptorchidism	11.28	4.58	4.48 × 10^–02^	8.27	NS	12.34^d^	↑
Nystagmus	9.75	3.82	NS	9.82^d^	NS	10.15^d^	–
Hearing impairment	9.38	8.40^d^	NS	13.18^d^	4.32 × 10^–02^	8.68	–
Cleft palate	9.26	6.87	NS	7.24	NS	9.83^d^	–
Failure to thrive	8.97	3.82	NS	11.89^d^	NS	8.73	–
***Top 15 HPO terms for the borderline-common disorders***							
Seizures	22.92^d^	16.79	NS	23.77^d^	NS	23.17^d^	–
Arthralgia	3.33	12.21	2.22 × 10^–06^	9.82^d^	7.92 × 10^–12^	1.41	↓
Depressivity	2.72	10.69	1.06 × 10^–05^	6.98	2.88 × 10^–07^	1.31	↓
Short stature	21.40^d^	10.69	1.14 × 10^–02^	17.31^d^	NS	22.96^d^	↑
Abdominal pain	2.84	9.92	2.07 × 10^–05^	8.53	2.25 × 10^–10^	1.20	↓
Fatigue	3.87	9.92	8.42 × 10^–04^	10.34^d^	1.13 × 10^–09^	2.14	–
Headache	2.35	9.92	4.11 × 10^–05^	4.65	1.68 × 10^–03^	1.36	↓
Hypertension	3.74	9.92	1.36 × 10^–03^	8.79	7.87 × 10^–07^	2.30	↓
Hepatomegaly	5.64	9.16	NS	11.11^d^	2.64 × 10^–05^	4.29	–
Renal insufficiency	3.13	9.16	8.77 × 10^–04^	7.49	2.62 × 10^–06^	1.83	↓
Attention deficit hyperactivity disorder	3.00	8.40	1.17 × 10^–02^	4.13	NS	2.41	↓
Constipation	3.54	8.40	4.88 × 10^–02^	8.27	2.98 × 10^–06^	2.25	↓
Diarrhea	2.51	8.40	1.37 × 10^–03^	4.91	3.82 × 10^–03^	1.62	↓
Hearing impairment	9.38^d^	8.40	NS	13.18^d^	4.32 × 10^–02^	8.68	–
Jaundice	1.85	8.40	2.34 × 10^–05^	4.91	7.99 × 10^–06^	0.78	↓
Muscle weakness	4.20	8.40	3.34 × 10^–02^	8.79	5.18 × 10^–05^	2.98	↓
Scoliosis	14.94^d^	8.40	NS	16.28^d^	NS	15.12^d^	–
Sleep disturbance	3.17	8.40	1.79 × 10^–02^	4.13	NS	2.62	↓
Splenomegaly	4.16	8.40	4.02 × 10^–02^	8.01	6.32 × 10^–04^	3.09	↓

Here, we focus on the top 15 HPO terms for the rare disorder (RD) spectrum and borderline-common disorders of which 2430 out of 3,524 RDs and 131 out of 145 RDs hold phenotypic information (equivalent to 69.0% and 90.3%), respectively. For the rare disorder category, 387 out of 412 RDs hold phenotypic information (93.9%), and for the ultra-rare disorder category, 1,912 out of 2,967 RDs hold phenotypic information (64.4%). The proportions within the borderline-common and rare disorder categories were compared with those within the ultra-rare disorder category

^a^Direction of change of proportions from borderline-common to ultra-rare (D)

^b^Proportion (prop) was calculated based on the number of RDs with phenotypic information within disorder category

^c^FDR-adjusted *p* < 0.05 was considered statistically significant; NS refers to not significant

^d^marks the HPO terms in top 15 within the disorder category

Focusing on the phenotypes observed for the RDs, HPO terms were assigned to 2430 out of the 3524 RDs forming the spectrum [38]. The top 5 HPO terms implicated in the RDs were seizures, intellectual disability, short stature, global developmental delay, and microcephaly (Table 3). Seizures and global developmental delay were highly co-occurrent (Fig. 5B); and global developmental delay was more often observed for RDs belonging to the rarer end of the spectrum (Table 3), which could reflect shared underlying pathophysiological mechanisms. Our categorization of HPO terms to the disorder categories borderline-common, rare and ultra-rare, and the HPO co-occurrence matrix can be found in Additional File 3. Subsets of the HPO co-occurrence matrix are visualized in Fig. 5B, C (enlarged in Additional File 1: Fig. S2-3).

Next, we conducted enrichment analyses of Reactome pathways among the disorder-associated genes from Orphadata [39] to learn more about how the phenotypes were manifested. This led to the identification of 26, 67 and 215 enriched pathways for the borderline-common, rare, and ultra-rare disorder category, respectively (Fig. 3A, Additional File 2: Table S3). Eleven enriched Reactome pathways overlapped between the three disorder categories (e.g., signal transduction, immune system, and developmental biology; Fig. 3C). Among the top 20 enriched Reactome terms for each of the disorder categories (Fig. 4), pathways such as “gene expression” (R-HSA-74160), “cell cycle” (R-HSA-1640170), “post-translational protein modification” (R-HSA-597592), “vesicle-mediated transport” (R-HSA-5653656), and “translation” (R-HSA-72766) were specific to the ultra-rare disorders.

For each disorder category, the majority of the RDs were classified as a disease (55.4–76.7%; Table 1). The proportion of RDs classified as a morphological anomaly decreased with rarity, whereas the proportion of RDs classified as a malformation syndrome (defined by Orphanet as disorders *“resulting from a developmental anomaly involving more than one morphogenetic field”* [36]) increased with rarity (Table 1). Moreover, most RDs in the spectrum were classified as a rare developmental defect during embryogenesis, rare neurologic disease, or rare inborn errors of metabolism (Table 1). A considerable proportion of ultra-rare disorders was classified as a rare developmental defect during embryogenesis, which is related to the high proportion of malformation syndromes.

### Some clinical characteristics are more prevalent among the borderline-common disorders than the rare and ultra-rare disorders

**Fig. 6:**
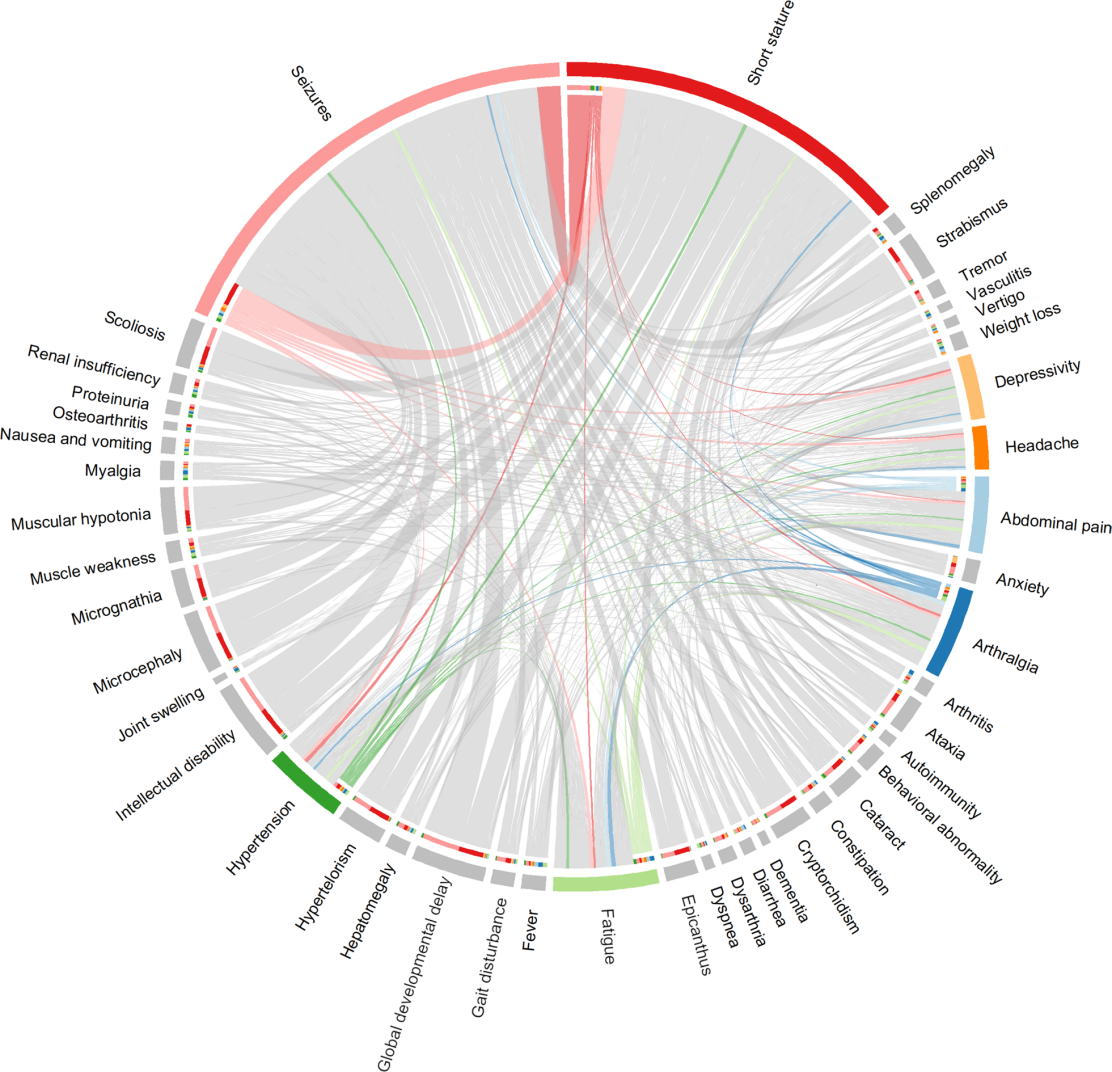
Occurrent Human Phenotype Ontology (HPO) terms for borderline-common disorders, and top 10 co-occurrent HPO terms. The following HPO terms prevalent for the borderline-common disorders were selected: Abdominal pain, arthralgia, fatigue, hypertension, seizures, short stature, depressivity, and headache (as presented in Table 3; highlighted using the selected color palette). For each of these HPO terms, we selected the most co-occurring HPO terms in the rare disorder (RD) spectrum (top 10, marked as grey) based on the number of RD that exhibit this pairwise combination of HPO terms. For example, seizures and global developmental delay can be observed for 234 RDs whereas seizures and hypertension only can be observed for 19 RDs. The more RDs exhibiting a certain combination of HPO terms, the thicker the line

In our study, we found that only two HPO terms (seizures and short stature) in top 15 for the entire RD spectrum overlapped with those in top 15 for the borderline-common disorder category (Table 3). Our findings indicate that phenotypes observed for the borderline-common disorders to some extent differ from the disorders belonging to the other categories (Table 2, Fig. 5). The borderline-common disorders have more HPO terms in common with the rare disorders and less with the ultra-rare disorders (Fig. 5A). HPO terms such as headache, depressivity, hypertension and sleep disturbance were more prevalent in the borderline-common disorders (Fig. 5A, Table 3), suggesting that patients presenting those phenotypes are more likely to be affected by a borderline-common type of disorder. Interestingly, by looking at the co-occurrence of HPO terms within the borderline-common category, depressivity tends to co-occur with anxiety, and headache with seizures but also fatigue, and nausea and vomiting (Fig. 5C, Additional File 1: Fig. S4). A subset of the HPO term co-occurrence matrix, selected based on phenotypes prevalent for the borderline-common disorders, can be found in Fig. 5C and Additional File 1: Fig. S4. On the contrary, HPO terms such as intellectual disability, short stature, global developmental delay, hypertelorism and strabismus were reported more often for rare and ultra-rare disorders proportion-wise (Table 3). Analysis of top HPO terms for the borderline-common disorders with their top 10 co-occurrent HPO terms can be found in Fig. 6.

In terms of enriched Reactome pathways, muscle contraction (R-HSA-397014) and cardiac conduction (R-HSA-5576891), both enriched in the three disorder categories, had significantly more annotated disorder-associated genes in relation to the total number of disorder-associated genes for the borderline-common disorder category as compared with the ultra-rare disorder category (Fig. 3C). When comparing the borderline-common and ultra-rare disorder categories, this was also the case for the overlapping Reactome pathways, axon guidance (R-HSA-422475), and striated muscle contraction (R-HSA-390522; Additional File 1: Table S4). Such differences were not observed for enriched Reactome pathways overlapping between the rare and ultra-rare disorder category (Additional File 1: Table S5).

### Patients with borderline-common disorders show more phenotypic variability

Variable expressivity has been described as *“the series of signs and symptoms that can occur in different people with the same genetic condition”* [40], and can confuse the diagnostic process. We developed the following two measures to reveal if the expressivity varied between the disorder categories: HPO term_obligate/very frequent_/HPO term_total_ where a lower estimate indicates more variable expressivity, and HPO term_occasional/very rare_/HPO term_total_ where a higher estimate indicates more variable expressivity (Table 2). The phenotypic expressivity for both the borderline-common and rare disorder category was significantly different from that of the ultra-rare disorder category. Here, the borderline-common disorders exhibited more variable phenotypic expressivity followed by the rare disorder category and then the ultra-rare disorder category (Table 2). Focusing solely on disorders with an oligogenic or multigenic/multifactorial inheritance, we found similar estimates as those for the borderline-common and/or rare disorder category [reported as median (IQR), HPO term_obligate/very frequent_/HPO term_total_ = 0.26 (0.36); HPO term_occasional/very rare_/HPO term_total_ = 0.40 (0.34)].

Even though clinical syndromes only comprise a small proportion of the RDs, more borderline-common disorders belonged to this disorder type proportion-wise compared with the rare and ultra-rare disorder categories (Table 1). A clinical syndrome has been described by Johnson et al. [41] as a *“constellation of clinical findings caused by an underlying disease(s) that may or may not be accompanied by laboratory or imaging abnormalities”*. To diagnose several clinical syndromes (e.g. acute lung injury [Orphacode:178320] [42], West syndrome [Orphacode:3451] [43] and TEMPI syndrome [Orphacode:284227] [44]), the practitioners only need the patient to present some features among the clinical diagnostic criteria—not all [45]. Interestingly, this might indicate that patients with the same clinical syndrome have slightly different clinical representations potentially coinciding with the higher proportion of borderline-common disorders having variable phenotypic expressivity. As only few clinical syndromes have associated genes (18.2% across the spectrum), we assume that those clinical syndromes are indeed challenging to resolve genetically.

## Discussion

Focusing on rare diseases in children, an average diagnostic rate of 42% has been reported when using trio-based whole-genome sequencing [4–6]. Considered the relatively low diagnostic rate, there is still a long way to go to ensure diagnostic success for those living with a rare disease. To improve the diagnostic success, we believe that we need to better understand the entire spectrum of rare disorders (acquired and congenital disorders) to tailor our diagnostic computational pipelines. Therefore, we characterized the RD spectrum using the disorder categories, borderline-common, rare, and ultra-rare. Searching through the literature, we were unable to find discussion of borderline-common disorders. Ultra-rare disorders, on the other hand, have been mentioned using various terms such as ultra-orphan, ultra-rare, extremely rare and very rare [46–48]. Based on our findings, borderline-common disorders can be described as (1) more often being inherited in a multigenic/multifactorial manner, (2) having more variable phenotypic expressivity, and (3) to some degree being distinct from the rare and ultra-rare disorders phenotypically and pathophysiologically. For example, some phenotypes are more often observed for borderline-common disorders than for ultra-rare disorders, and vice versa. There also seem to be a difference in the involvement of biological pathways. Interestingly, Boycott et al. [49] highlighted in 2017 that ultra-rare and unrecognized genetic diseases are contributors to bottlenecks in gene-discovery pipelines. Our study explores the possibility of borderline-common disorders being contributors to bottlenecks as well.

With regard to the rarity of disorders, we found more ultra-rare disorders than rare and borderline-common disorders. This agrees with Hennekam who reported that *“as a group they form a considerable part of the total group of persons with rare disorders in the European Community”* [50]. Nevertheless, this only collectively translates to 4.1% of individuals in a fictive cohort of RD patients living with an ultra-rare disorder (Fig. 2). The ultra-rare disorders were primarily associated with a single gene and inherited in either an autosomal recessive (42.5%) or autosomal dominant (25.5%) pattern. Another characteristic of ultra-rare disorders was that the phenotypic variability was less pronounced. This makes patients belonging to this category suitable for most diagnostic computational pipelines, and thus the diagnostic rate for this group of patients might be higher in comparison with those living with borderline-common disorders.

Implication of multiple genes, reduced penetrance, and variable expressivity is expected for the more common disorders within the RD spectrum, and are commonly not accounted for by traditional computational pipelines. We believe that applying individual patient-tailored methods, that account for more complex genetic and phenotypic scenarios, will improve those diagnostic rates. We know that incomplete penetrance and variable expressivity affect the correlation between the genotype and phenotype [11], which complicates the diagnostic process, especially when diagnosing more common RDs. Here, we refer to those RDs that are more common on a global scale (not to be confused with RDs that have become common in specific population/region). We observed more variable phenotypic representations for disorders within the borderline-common disorder category using our newly constructed phenotypic expressivity measures (based on the HPO terms and their occurrence). This most likely coincide with our finding that a higher proportion of borderline-common disorders are inherited in a multigenic/multifactorial pattern. You might ask, *‘but why do you care about borderline-common disorders when they only comprise 4.1% of RDs within the spectrum?’* By considering the number of RDs together with the point prevalence of those disorders, the majority of individuals with a disorder belonging to the RD spectrum in a fictive cohort most likely fall into the borderline-common category (more than 90%; Fig. 2). So, due to the construct of today’s pipelines, the higher prevalence of borderline-common disorders, combined with their more complex genetic and phenotypic scenarios, might result in more of these patients ending up in the ‘difficult-to-diagnose’ or ‘undiagnosed’ category. This also seems to go hand in hand with our findings that one RD within the rare disorder category is caused by oligogenic inheritance, namely Bardet-Biedl syndrome [Orphacode:110]. Several studies are available which focus on the inheritance patterns of this syndrome, including oligogenic inheritance (e.g. triallelic inheritance has been observed) [51]. In this context, oligogenic inheritance was defined as occurring *“when specific alleles at more than one locus affect a genetic trait by causing and/or modifying the severity and range of a phenotype”* [51]. We expect that the number of RDs, linked to this inheritance type, will increase over time due to the exploration of more complex genetic scenarios. Moreover, as the number of disorder-associated genes increase with decreasing rarity focus on susceptibility factors and modifiers in future pipelines might help us to better understand underlying genetic architecture and why variable phenotypic expressivity can be observed for a wide range of rare disorders. This was discussed by Rahit and Tarailo-Graovac in 2020 [21].

Not only does the phenotypic expressivity tend to vary across RD spectrum, but the same happens with the palette of phenotypes too. When assessing the phenotypes associated with the borderline-common disorders, they tended to differ from those found for the rare and ultra-rare disorders. When interpreting this finding, one needs to take into consideration that some patients with the rarer disorders might exhibit communication difficulties, and so, might be unable to express if they are feeling depressed or having a headache (phenotypes more common among the borderline-common disorders). On the other hand, these findings could also be indicative of variable essentiality of mutated genes and/or implication of variable developmental phases. As we have not been able to find any literature on this research area, we have not compared it with current knowledge. Nevertheless, it is recognized that use of high-dimensional phenotypic profiles can be one way to improve diagnostic success. For example, Turro and colleagues were able to genetically diagnose 16.1% of the patients (n = 7065) with extensive phenotypic profiles [52]. Interestingly, in the initial phase of this study, we tried to perform a cluster analysis focusing on the HPO terms to learn more about the RDs in the spectrum (clustering RDs based on their phenotypic profile). This turned out to be more challenging than expected. For example, only a considerably small variation between RDs was explained for each dimension (clustering of multidimensional data). It could indicate presence of high complexity for the disorders within the RD spectrum and thus a combination of phenotypes, genes and biological pathways might be beneficial. In 2021, the RD map was made publicly available, which utilizes a combination of HPO and GO terms to construct their network of rare diseases [53].

Discovery of genes associated with RDs has steadily improved in the years since next-generation sequencing became available [1]. As our study is based on information stored in publicly available databases, we explored what is already known about RDs and disorder-associated genes (ranging from disorder-causing to modifying genes). Our findings are of direct relevance to undiagnosed RD patients that fall into an already established diagnosis (‘the diagnosis phase’). Yet, by extrapolating the findings within the entire RD spectrum, we believe our study to also have relevance for patients falling outside an established diagnosis (‘the discovery phase’). When searching for underlying causes of novel rare disorders, one might find that mutations causing these disorders are located in genes belonging to the same gene–gene/protein–protein networks or biological pathways as the mutated genes known to cause already known disorders with similar phenotypic representations; this trend has been observed in the past [54].

We expect that tailoring of our in-house computational pipeline to account for more complex genetic and phenotypic scenarios might help to elucidate the underlying cause of disease in the Indigenous patients enrolled in Silent Genomes who remain undiagnosed after our level 1 analysis (‘state-of-the-field’ approaches looking mainly for single genes defects in the form of SNVs and some types of structural variants). In the case that the level 1 analysis fails, the patients are moved to level 2 analysis where we create and utilize novel approaches. This will allow for applying different, specifically built, and individual patient-tailored methods in cases for which, we expect that variants from multiple genes are involved in the disease etiology or reduced penetrance and variable expressivity is suspected (Silent Genomes is currently not powered to investigate more complex genetic and phenotypic scenarios).

## Conclusions

Our initial goal was to enable the answering of the following question: *How can we distinguish between the disorder categories, borderline-common, rare, and ultra-rare?* We learned that the disorder categories can be described by a wide range of factors, including disorder types, linearisation parents, biological pathways, and phenotypes, which we now can use to categorize undiagnosed patients into a specific disorder category. For example, if an undiagnosed patient with a RD gets assigned to the borderline-common disorder category, one might suspect that more than one gene could be involved in the etiology of the disorder, and thus the pipeline chosen should be able to address more complex genetic scenarios. So, we can use the findings presented in the current study to choose the most appropriate statistical methodology for the patient in question to improve diagnostic success but also to learn more about specific populations. Finally, understanding of the involvement of pathophysiological mechanisms for each of the disorder categories can potentially help us to pinpoint what genes might be causative for the undiagnosed patient in question and it could be useful in narrowing down the list of genetic variants outputted from our computational pipeline.

## Methods

In this study, we characterized the RD spectrum in silico by conducting bioinformatics analyses focusing specifically on *Homo Sapiens*, and the following disorder categories: Borderline-common, rare and ultra-rare.

### Data extraction from Orphanet and filtering

Data were extracted from Orphadata (i.e., publicly available datasets underlying the rare disease database Orphanet) on June 1, 2020, which included information about epidemiology, associated genes and phenotypes, natural history and linearisation of disorders (monohierarchical view of classified disorders referred to as linearisation parents) [37]. The data was processed using the statistical software R (version 4.0.2). After combining the separate datasets, twelve variables of interest were available (names, synonyms, Orphacodes, prevalence, disorder groups, disorder types, linearisation parents, type of inheritance, interval average age of onset, interval average age of death, associated Human Phenotype Ontology (HPO) terms [38], and associated genes). The reported type(s) of inheritance was based on the literature and the Orphanet encyclopedia (e.g. expert reviews for creation and updating of disorder summary texts) and associated genes on available peer-reviewed publications. An overview of the data availability across disorder types can be found in Additional File 1: Table S1. Subsequently, RDs were filtered as follows: (1) RDs with known prevalence were selected, (2) RDs categorized as ‘group of disorders’ and ‘subtype of disorders’ were excluded, and (3) RDs belonging to one of the linearisation parents ‘rare disorder due to toxic effects’, ‘rare infectious disease’ or ‘rare neoplastic disease’, were excluded. Further details on certain inclusion and exclusion criteria can be found in Nguengang Wakap et al. (2020). Finally, only RDs with known worldwide and/or continent point prevalence were included in the study (Additional File 1: Fig. S1).

### Assigning point prevalence and disorder category

To provide additional context, measures of disorder frequency were reported as point prevalence, birth prevalence and annual incidence in Orphadata [37]. Regarding specific categories, six prevalence categories were available, namely > 1/1,000, 6–9/10,000, 1–5/10,000, 1–9/100,000, 1–9/1,000,000 and < 1/1,000,000. As most sources investigated point prevalence, this was the type of disease frequency we decided to focus on in our study. When assigning prevalence categories to the RDs, worldwide point prevalence was prioritized over continent point prevalence. For those RDs with unknown worldwide point prevalence, continent point prevalence was used and the prevalence category which had been reported most times was assigned. If multiple prevalence categories were reported an equal number of times (this only occurred for 4 RDs), we assigned the prevalence category reported in the user-friendly online version, Orphanet [36]. Afterwards, the RDs were assigned to one of the following disorder categories based on their point prevalence: Borderline-common (6–9/10,000 and 1–5/10,000), rare (1–9/100,000 and 1–9/1,000,000) and ultra-rare (< 1/1,000,000) (Additional File 1: Fig. S1; by using broad point prevalence categories, we do not account for the variability within the disorder categories). To gain further insight into the frequency at which the disorder categories can be observed in a population, we created a fictive cohort consisting of 500 individuals. The proportions at which the disorder categories can be observed in a population were calculated as follows: Point prevalence (midpoint) * individuals in fictive population * number of RDs * 100. It is also important to consider that, on one hand, the ultra-rare disorder category might result in more precise estimates due to the considerable number of RDs that falls into this category. On the other hand, more patients have a borderline-common disorder which could improve the estimate precision too.

### Phenotypes and genes across the rare disorder spectrum

RDs with HPO information were selected. The occurrence of the HPO terms was reported as follows in Orphadata: ‘Obligate (100%)’, ‘very frequent (99–80%)’, ‘frequent (79–30%)’, ‘occasional (29–5%)’, ‘very rare (< 4–1%)’ and ‘excluded (0%)’. The occurrence category ‘excluded (0%)’ was omitted. In addition to counting HPO terms with certain occurrences, following ratios were calculated for each RD in the spectrum: (1) Ratio between the number of obligate and very frequent HPO terms and the number of all HPO terms, and (2) ratio between the number of occasional and very rare HPO terms and the number of all HPO terms for the RD in question. The ratios were used as measures of expressivity, *“the phenomenon of differing clinical features or phenotype among individuals carrying the same gene allele or genotype”* [55]. Moreover, we constructed a co-occurrence matrix for HPO terms across the RD spectrum revealing for how many RDs each pairwise combination of HPO terms co-occur. Subsets of the co-occurrence matrix (selected based on occurrent HPO terms within the RD spectrum and borderline-common disorder category) were visualized using heatmaps with dendrograms by utilizing the gplots R package [56]. Moreover, the top HPO terms for the borderline-common disorders and their most co-occurrent HPO terms (top 10) were selected. The co-occurrence between the selected HPO terms were visualized using the circlize R package [57].

For each list of associated genes (from the Orphadata) within the disorder categories, we conducted enrichment analyses of Reactome pathways (version 65; investigates if the genes found for a specific disorder category are enriched for any Reactome pathway) using the PANTHER [Protein ANalysis THrough Evolutionary Relationships] database [58, 59]. The enrichment analyses were conducted using Fisher’s exact tests and the computed *p-values* were adjusted using the false discovery rate (FDR) method. *p* < 0.05 was considered statistically significant. Additionally, we only considered enriched terms with at least 10 annotated genes (or gene products) associated with the disorder category as being truly enriched.

### Significance testing of proportions and distributions

For the variables of interest, we measured proportions, medians, and interquartile ranges (IQR). We tested whether the proportions and distributions for the borderline-common and rare disorder categories differed from the ultra-rare disorder category. As the sample size for some variables was small, Fisher’s exact tests were used to test differences in proportions, and two-tailed *p-values* were computed and adjusted for multiple testing (FDR < 0.05). For the continuous variables, we tested the null hypothesis (H_0_: the distribution parameters are the same in each group) against the alternative hypothesis (H_A_: the distribution parameters are not the same in at least one group) to reveal if there was a significant difference between the mean ranks of the disorder categories, borderline-common, rare and ultra-rare. This was done using the Kruskal–Wallis Rank Sum test. If significant differences were observed between disorder categories, pairwise comparisons were conducted using Pairwise Wilcoxon Rank Sum tests. The computed *p-values* were adjusted for multiple testing using the FDR method, as for the proportions.

An overview of the methodological workflow can be found in Additional File 1: Fig. S2.

## Supplementary Information

**Additional file 1**. This .pdf file contains supplementary figures and tables (Figures S1–S5, Tables S1, S4 and S5). Figure S1. Orphadata and related study flowchart. Figure S2. Methodology and related study flowchart. Figure S3. Human Phenotype Ontology term co-occurrence for the rare disorder spectrum. Figure S4. Human Phenotype Ontology term co-occurrence focusing on the borderline-common disorders. Figure S5. Distributions of Human Phenotype Ontology term and disorder-associated gene counts. Table S1. Disorder types and data availability. Table S4. Enriched Reactome pathways in borderline-common and ultra-rare disorders. Table S5. Overrepresented Reactome pathways for the rare and ultra-rare disorders.

**Additional file 2**. This .xlsx file contains supplementary tables (Tables S2–S3). Table S2. Overview of borderline-common disorders based on Orphadata and our predefined criteria. Table S3. Overview of the enriched Reactome pathways for each of the disorder categories, borderline-common, rare, and ultra-rare disorder category, focusing specifically on Homo Sapiens genes.

**Additional file 3**. This RAR compressed .xlsx file contains our categorization of HPO terms to the disorder categories, borderline-common, rare and ultra-rare, and the HPO co-occurrence matrix for the RD spectrum.

## Data Availability

The results presented in this paper were obtained using the source data (orientdb version) for Orphanet available via the site http://www.orphadata.org/, combined with other web resources (https://www.orpha.net/ and http://www.pantherdb.org/).

## Abbreviations

DECIPHER: DatabasE of genomiC varIation and Phenotype in Humans using Ensembl Resource
DIDA: DIgenic diseases Database
FDR: False discovery rate
gnomAD: Genome aggregation database
HPO: Human Phenotype Ontology
IQR: Interquartile ranges
ORVAL: Oligogenic Resource for Variant AnaLysis
PANTHER: Protein ANalysis THrough Evolutionary Relationships
RD: Rare disorder
