# Supplementary material for: Rare disorders have many faces: in silico characterization of rare disorder spectrum
Source: Orphanet J Rare Dis. 2022 Feb 22;17:76. doi: 10.1186/s13023-022-02217-9 (PMC8864832; doi:10.1186/s13023-022-02217-9)

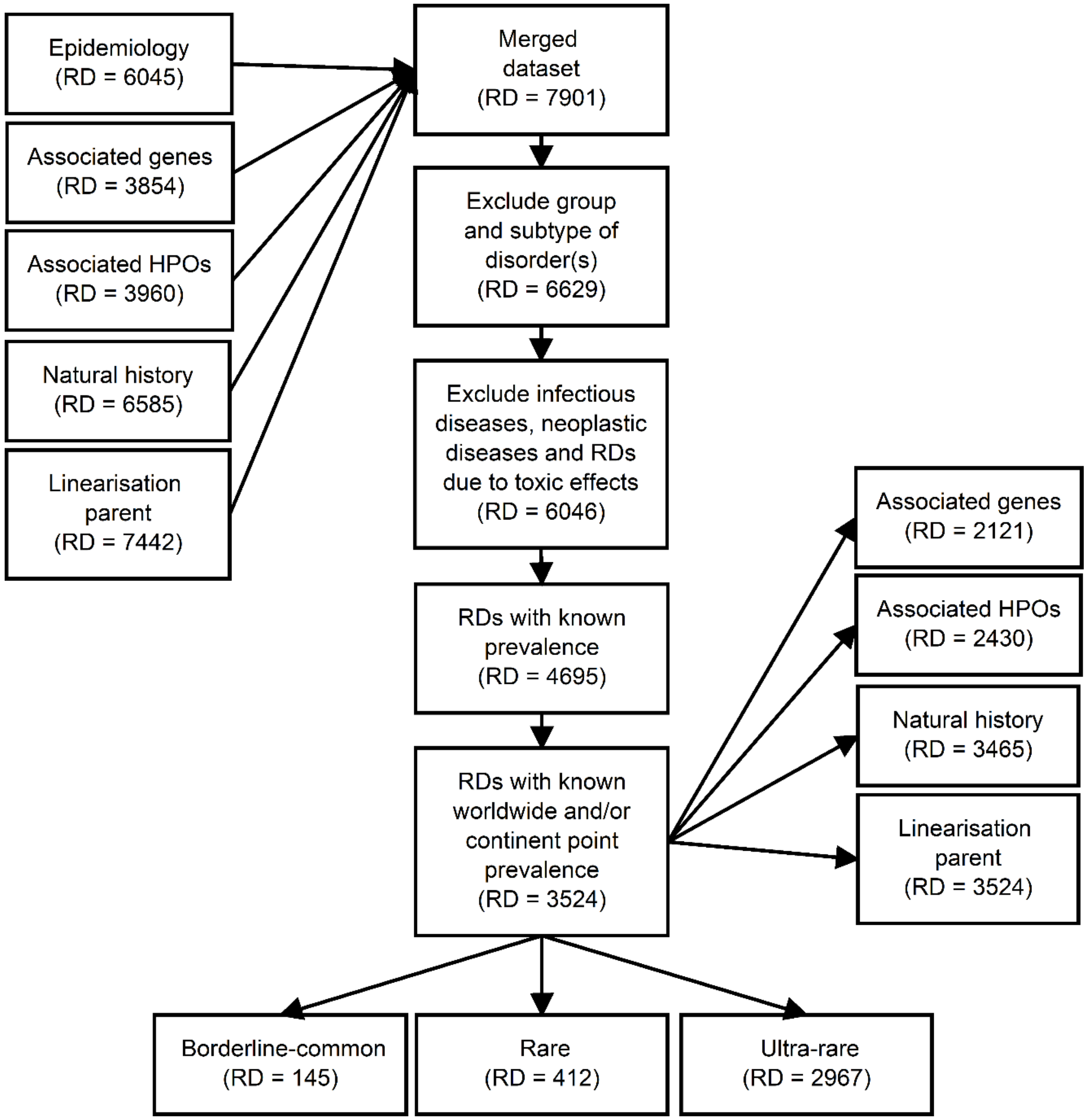

Fig. S1: Orphadata and related study flowchart. The flowchart displays the steps from the raw aggregated datasets from Orphadata[32] to categorization of RDs to specific disorder categories. Orphadata and other sources were used to define the disorder categories within the rare disorder (RD) spectrum, namely borderline-common (6-9 / 10 000 and 1-5 / 10 000), rare (1-9 / 100 000 and 1-9 / 1 000 000) and ultra-rare (< 1 / 1 000 000), and to characterize the RD spectrum (methodological flowchart shown in Figure S2). RD prevalence is affected by various factors, including type of inheritance, number of associated genes, population structure (founder and consanguinity effects), population lifestyle and natural selection (differential survival and reproduction). *Abbreviations: HPO, human phenotype ontology; RD, rare disorder.*

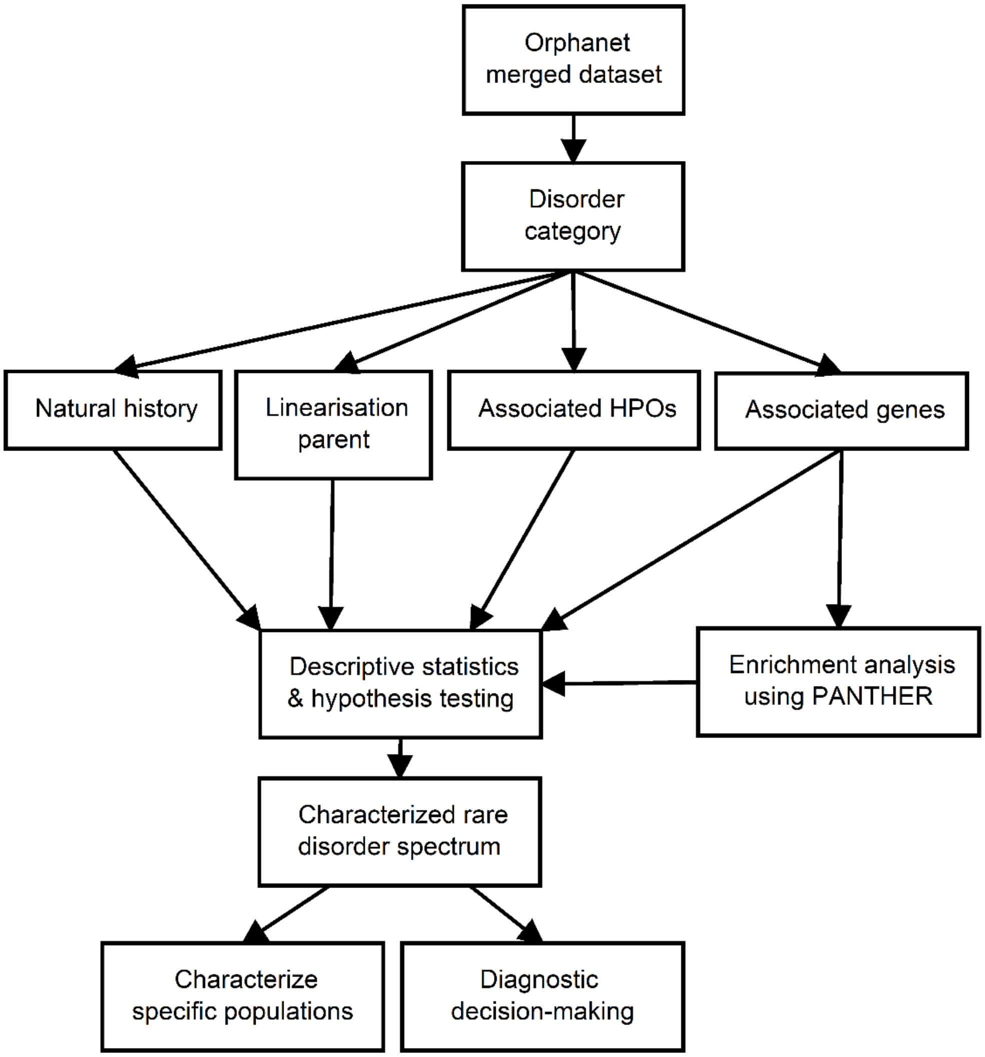

Fig. S2: Methodology and related study flowchart. The flowchart displays the steps from raw aggregated datasets from Orphadata to the characterization of the rare disorder spectrum. The characterized rare disorder spectrum can be used to characterize specific populations and for diagnostic decision-making in the future. *Abbreviations: HPO, Human Phenotype Ontology; PANTHER, Protein ANalysis THrough Evolutionary Relationships.*

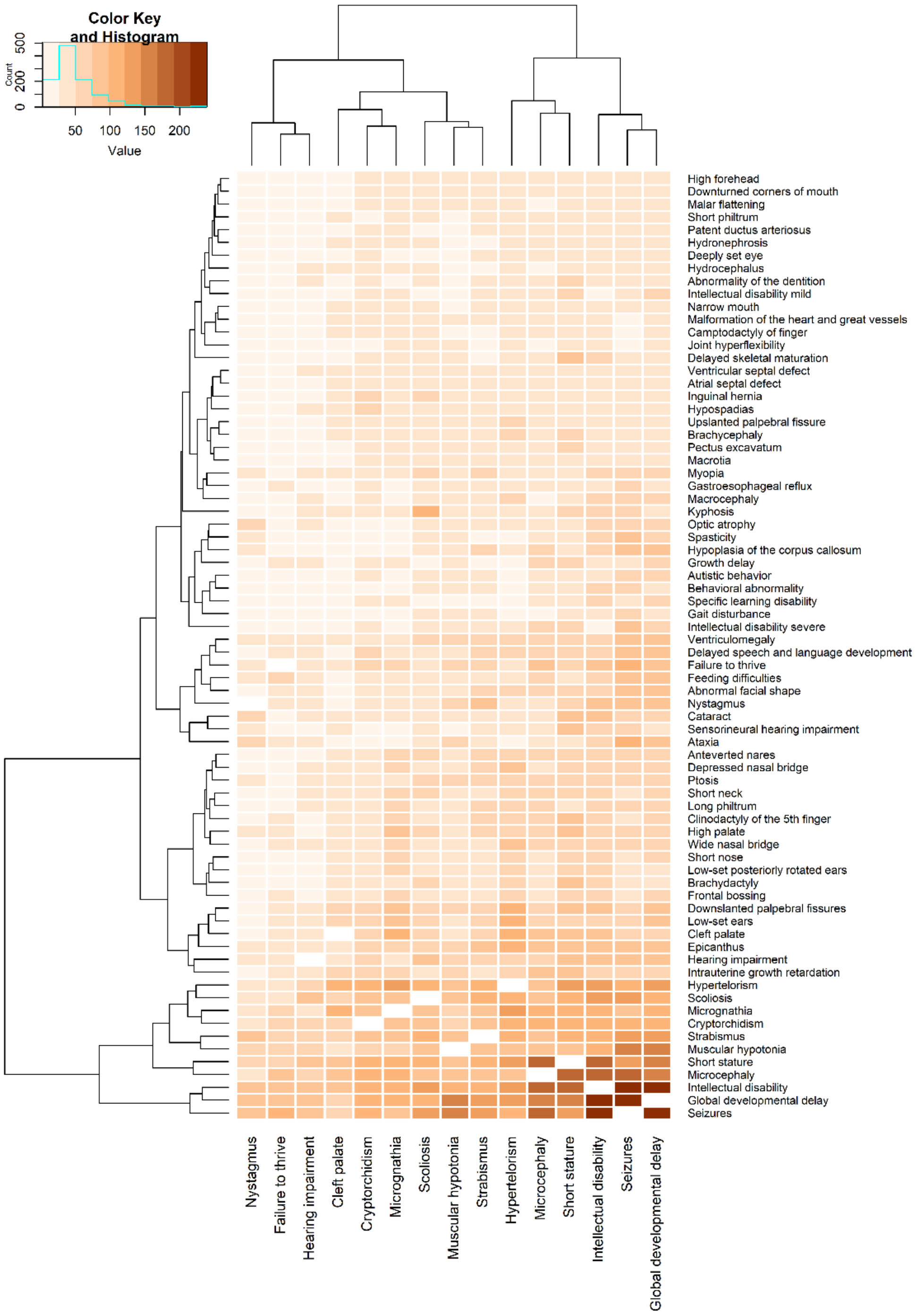

Fig. S3: Human Phenotype Ontology term co-occurrence for the rare disorder spectrum. Overview of the top 15 Human Phenotype Ontology (HPO) terms for the rare disorder spectrum (presented in Table 2) and their co-occurrence with a selection of HPO terms. These HPO terms were selected based on high relative co-occurrence. The HPO terms were clustered based on their co-occurrence profiles.

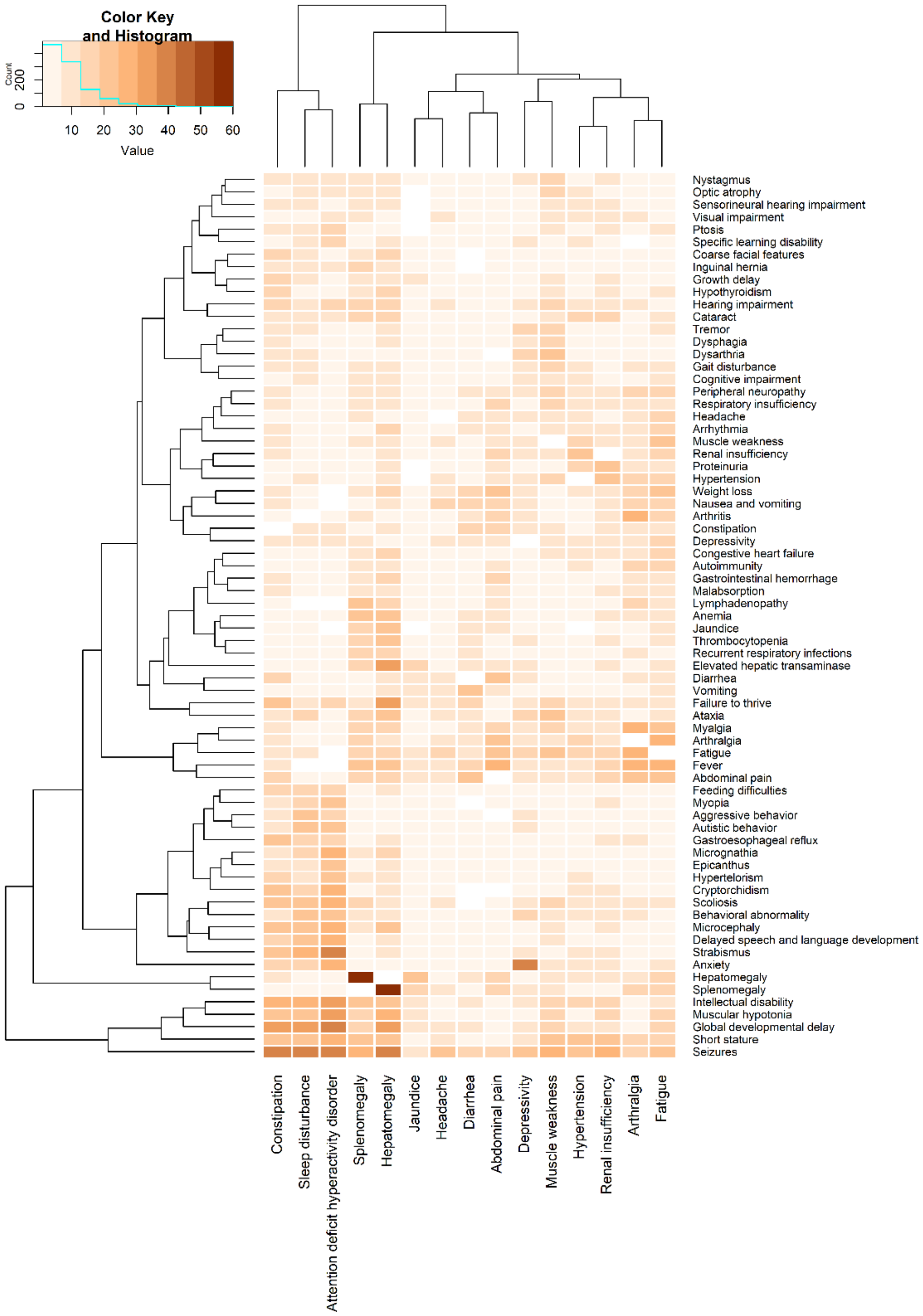

Fig. S4: Human Phenotype Ontology term co-occurrence focusing on the borderline-common disorders. Overview of the top 15 Human Phenotype Ontology (HPO) terms for the borderline-common disorder category and their co-occurrence with a selection of HPO terms. These HPO terms were selected based on high relative co-occurrence. The HPO terms were clustered based on their co-occurrence profiles. Here, we excluded the HPO terms, hearing impairment, seizures, short stature, and scoliosis, from top 15 as they are represented in Figure S2.

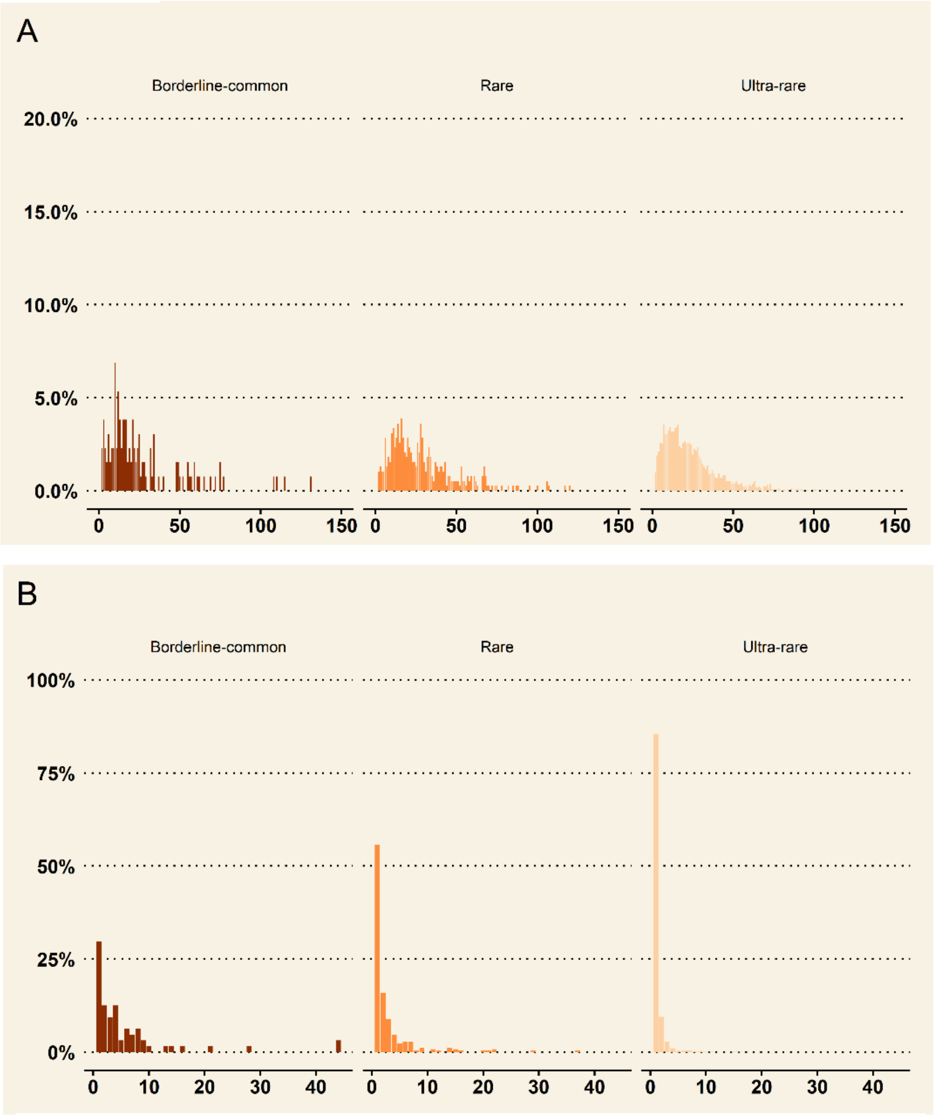

Fig. S5: Distributions of Human Phenotype Ontology term and disorder-associated gene counts. A. Distribution of the total number of Human Phenotype Ontology terms per disorder. B. Distribution of the total number of disorder-associated genes per disorder.

Table S1: Disorder types and data availability. Here is an overview of the data available for each of the disorder types within the rare disorder (RD) spectrum focusing on the following variables: Linearisation parent, age of onset, age of death, type of inheritance, associated Human Ontology Phenotype (HPO) terms, and disorder-associated genes.

**Disorder types**	**Linearisation parent**	**Age of onset**	**Age of death**	**Type of inheritance**	**Associated HPO terms**	**Associated genes**
Measure	Prop, %					
Disease (RD = 2049)	100.00	96.88	62.76	89.56	63.54	73.60
Malformation syndrome (RD = 1345)	100.00	97.03	68.33	76.73	78.29	42.90
Morphological anomaly (RD = 83)	100.00	97.59	55.42	79.52	67.47	36.14
Particular clinical situation in a disease or syndrome (RD = 31)	100.00	32.26	12.90	12.90	25.81	0.00
Clinical syndrome (RD = 11)	100.00	72.73	45.45	63.64	90.91	18.18
Biological anomaly (RD = 5)	100.00	100.00	80.00	60.00	20.00	80.00

Table S4: Enriched Reactome pathways in borderline-common and ultra-rare disorders. Overview of enriched Reactome pathways that overlap between the borderline-common and ultra-rare disorder categories, other than those presented in Figure 3C. The proportions within the borderline-common and rare disorder categories were compared with those within the ultra-rare disorder category. Significant differences were observed when comparing the proportion of genes annotated to the enriched Reactome pathways between borderline-common and ultra-rare disorders, but not between rare and ultra-rare disorders (Table S5).

Term	ID	Borderline-common			Ultra-rare	
		FE	Prop, %	Adj. p-value[a]	FE	Prop, %
Axon guidance	R-HSA-422475	2.94	7.62	2.0×10^{-02}	1.65	4.23
Hemostasis	R-HSA-109582	2.22	7.04	NS	1.94	6.10
Neuronal System	R-HSA-112316	2.76	5.28	NS	2.01	3.79
Striated Muscle Contraction	R-HSA-390522	29.22	4.99	6.1×10^{-06}	4.56	0.77
FLT3 Signaling	R-HSA-9607240	3.96	4.69	1.4×10^{-02}	1.73	2.03
L1CAM interactions	R-HSA-373760	8.39	4.69	1.9×10^{-03}	2.78	1.54
MAPK family signaling cascades	R-HSA-5683057	3.59	4.69	2.1×10^{-02}	1.70	2.20
Ion channel transport	R-HSA-983712	4.06	3.52	3.3×10^{-02}	1.79	1.54
Phase 0 - rapid depolarisation	R-HSA-5576892	16.87	3.52	5.3×10^{-04}	3.20	0.66
Platelet activation, signaling and aggregation	R-HSA-76002	2.87	3.52	NS	2.49	3.02
Interaction between L1 and Ankyrins	R-HSA-445095	21.95	3.23	1.2×10^{-03}	4.54	0.66
Ion homeostasis	R-HSA-5578775	11.46	2.93	1.0×10^{-02}	3.47	0.88

[a] FDR-adjusted $p < 0.05$ was considered statistically significant; NS refers to not significant.

Table S5: Overrepresented Reactome pathways for the rare and ultra-rare disorders. An overview of overrepresented Reactome pathways overlapping between the rare and ultra-rare disorder categories, other than those presented in Figure 3C. The proportions within the rare disorder category were compared with those within the ultra-rare disorder category. No significant differences were observed when comparing the proportion of genes annotated to the overrepresented Reactome pathways between rare and ultra-rare disorders.

Term	ID	Rare			Ultra-rare	
		FE	Prop, %	Adj. p-value*	FE	Prop, %
Metabolism	R-HSA-1430728	1.90	18.41	NS	1.96	19.12
Generic Transcription Pathway	R-HSA-212436	1.56	8.69	NS	1.30	7.31
Innate Immune System	R-HSA-168249	1.66	8.54	NS	1.43	7.42
Adaptive Immune System	R-HSA-1280218	1.74	6.63	NS	1.44	5.55
Signaling by Receptor Tyrosine Kinases	R-HSA-9006934	2.84	6.04	NS	2.23	4.78
Organelle biogenesis and maintenance	R-HSA-1852241	4.12	5.60	NS	2.49	3.41
Signaling by Interleukins	R-HSA-449147	2.55	5.30	NS	1.86	3.90
DNA Repair	R-HSA-73894	2.97	4.27	NS	1.82	2.64
Degradation of the extracellular matrix	R-HSA-1474228	4.75	3.09	NS	3.18	2.09
Disorders of transmembrane transporters	R-HSA-5619115	3.50	2.80	NS	3.34	2.69
Metabolism of vitamins and cofactors	R-HSA-196854	3.23	2.80	NS	2.39	2.09
Diseases of glycosylation	R-HSA-3781865	3.25	2.21	NS	4.42	3.02
Signaling by Hedgehog	R-HSA-5358351	3.23	2.21	NS	3.19	2.20
Signaling by MET	R-HSA-6806834	6.24	2.21	NS	3.86	1.37
DNA Double-Strand Break Repair	R-HSA-5693532	2.99	2.06	NS	2.14	1.48
ECM proteoglycans	R-HSA-3000178	5.83	2.06	NS	4.47	1.59
SUMO E3 ligases SUMOylate target proteins	R-HSA-3108232	2.82	2.06	NS	1.94	1.43
SUMOylation	R-HSA-2990846	2.72	2.06	NS	1.87	1.43
MHC class II antigen presentation	R-HSA-2132295	3.29	1.91	NS	2.25	1.32
Anchoring of the basal body to the plasma membrane	R-HSA-5620912	3.96	1.77	NS	3.78	1.70
Assembly of collagen fibrils and other multimeric structures	R-HSA-2022090	6.33	1.77	NS	4.69	1.32
Collagen degradation	R-HSA-1442490	5.93	1.77	NS	3.85	1.15
Collagen formation	R-HSA-1474290	4.27	1.77	NS	3.82	1.59
HDR through Homologous Recombination	R-HSA-5685942	5.75	1.77	NS	2.66	0.82
Integration of energy metabolism	R-HSA-163685	3.55	1.77	NS	2.85	1.43
Non-integrin membrane-ECM interactions	R-HSA-3000171	6.44	1.77	NS	4.77	1.32
Hedgehog 'off' state	R-HSA-5610787	3.14	1.62	NS	3.38	1.76
Interleukin-12 family signaling	R-HSA-447115	6.22	1.62	NS	2.93	0.77
Metabolism of water-soluble vitamins and cofactors	R-HSA-196849	2.90	1.62	NS	2.05	1.15
Signaling by PDGF	R-HSA-186797	6.45	1.62	NS	3.69	0.93
Signaling by VEGF	R-HSA-194138	3.38	1.62	NS	2.84	1.37
SLC transporter disorders	R-HSA-5619102	3.63	1.62	NS	4.40	1.98
VEGFA-VEGFR2 Pathway	R-HSA-4420097	3.70	1.62	NS	3.12	1.37
Integrin cell surface interactions	R-HSA-216083	3.77	1.47	NS	2.79	1.10
MET promotes cell motility	R-HSA-8875878	7.91	1.47	NS	5.27	0.99
NCAM signaling for neurite out-growth	R-HSA-375165	5.36	1.47	NS	2.78	0.77
Regulation of insulin secretion	R-HSA-422356	4.06	1.47	NS	3.16	1.15

* FDR-adjusted $p < 0.05$ was considered statistically significant; NS refers to not significant.

CHECK OUT THE CO-OCCURRENCE OF HPO TERMS IN THE RARE DISORDER SPECTRUM

Instructions

1. Open the ‘supplemental_data_HPO_cooccurrence.xlsm’ file
   (it is a large file, so it can take some time)
2. Filter the list of HPO terms by clicking on the arrow shown below, type in the keyword of your interest and click OK.

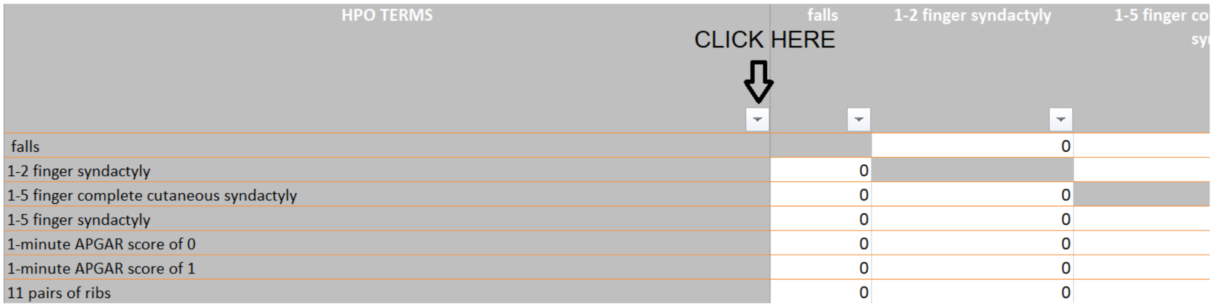

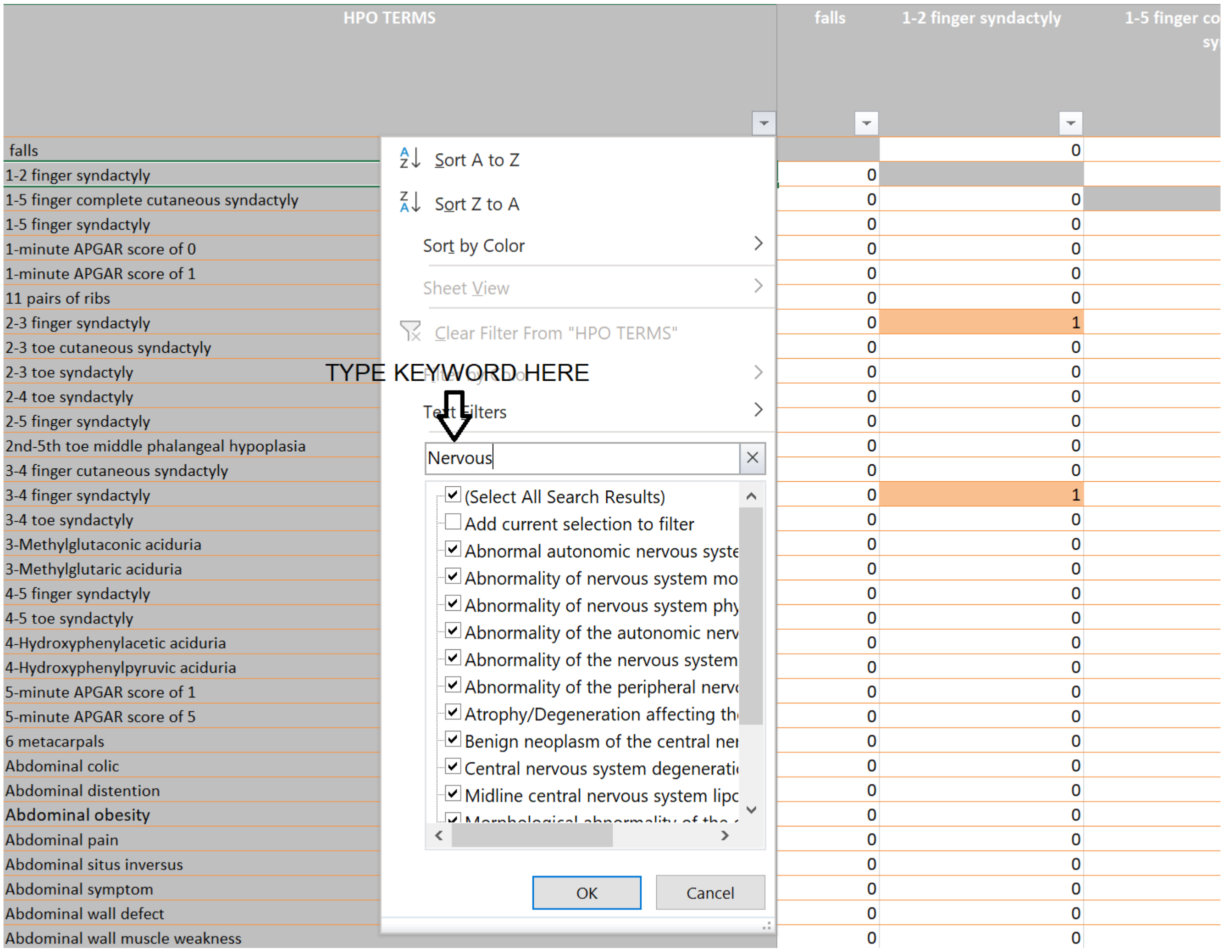

3. After the filtering, two buttons will appear. Click on the ‘HIDE COLUMNS WHEN COLUMN = 0’. This will give you only the columns that is of interest in regard to your selected HPO terms. This step will take about 1.5 min.

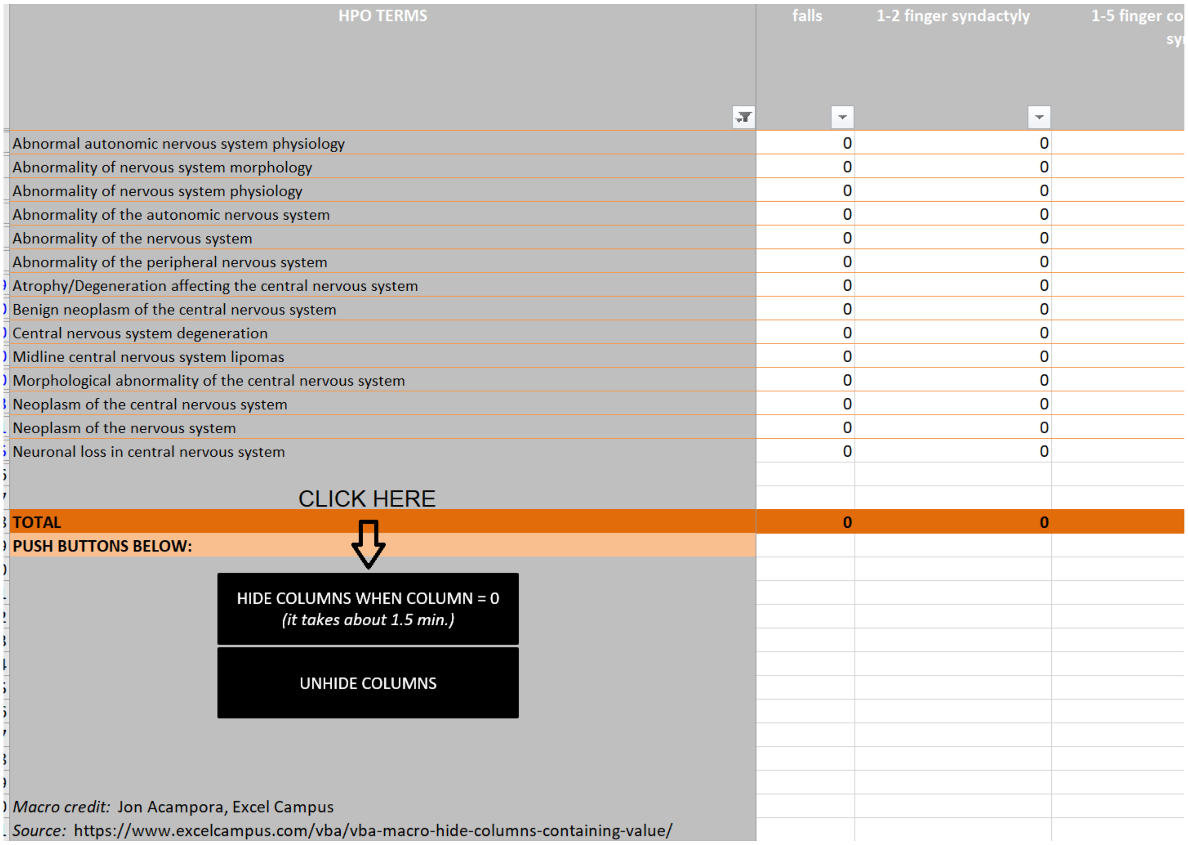

OBS: Remember to Enable Content before pushing the button

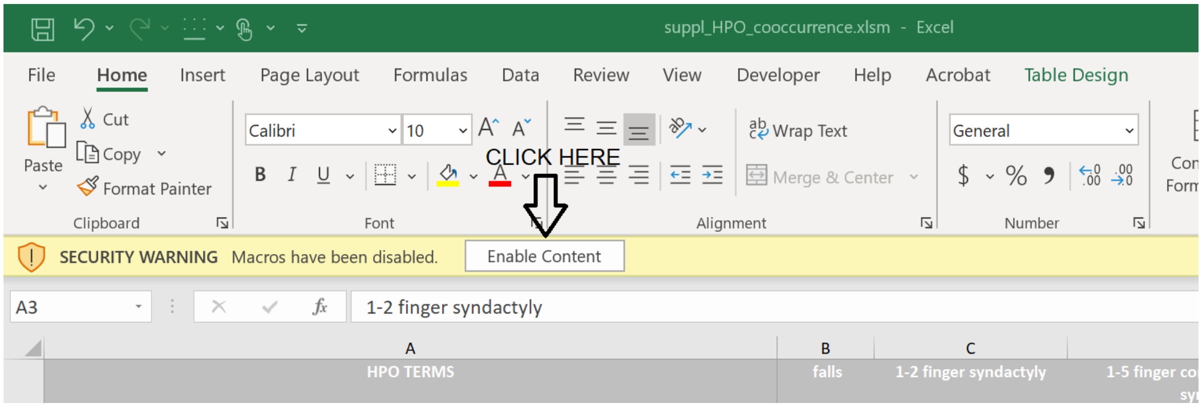

4. To start over, click on the 'UNHIDE COLUMNS' button and then 'Clear Filter From "HPO TERMS"

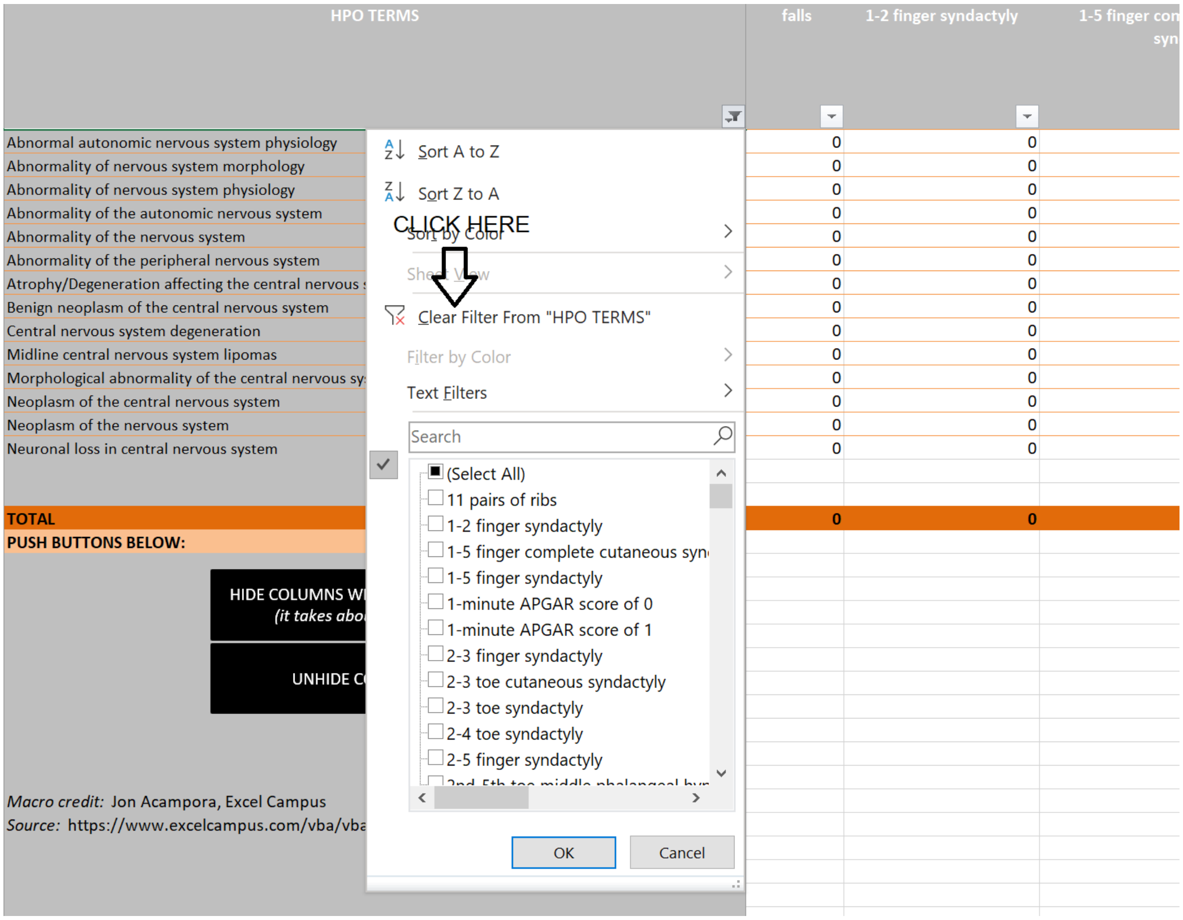

Supplement: Supplementary file 1 — Additional file 1. This .pdf file contains supplementary figures and tables (Figures S1–S5, Tables S1, S4 and S5). Figure S1. Orphadata and related study flowchart. Figure S2. Methodology and related study flowchart. Figure S3. Human Phenotype Ontology term co-occurrence for the rare disorder spectrum. Figure S4. Human Phenotype Ontology term co-occurrence focusing on the borderline-common disorders. Figure S5. Distributions of Human Phenotype Ontology term and disorder-associated gene counts. Table S1. Disorder types and data availability. Table S4. Enriched Reactome pathways in borderline-common and ultra-rare disorders. Table S5. Overrepresented Reactome pathways for the rare and ultra-rare disorders. [file 13023_2022_2217_MOESM1_ESM.pdf]
